# Supplementary material for: Perinatal factors and hospitalisations for severe childhood infections: a population-based cohort study in Sweden
Source: BMJ Open. 2021 Oct 7;11(10):e054083. doi: 10.1136/bmjopen-2021-054083 (PMC8499334; doi:10.1136/bmjopen-2021-054083)
Supplement: Supplementary data [file bmjopen-2021-054083supp001.pdf]

Appendix A

Table A: Incidence overall and for categories of bacterial meningitis in early childhood (28 days until 2 years of age), by year of birth

	1997–2002	2003–2008	2009–2013
	Incidence (95% CI)	Incidence (95% CI)	Incidence (95% CI)
All	16.6 (14.0–19.7)	19.6 (17.0–22.6)	9.0 (7.2–11.1)
Meningococcal meningitis*	2.5 (1.6–3.9)	1.6 (0.9–2.6)	1.2 (0.7–2.1)
Haemophilus meningitis†	1.0 (0.5–2.0)	1.0 (0.6–1.9)	0.7 (0.4–1.6)
Pneumococcal meningitis‡	8.3 (6.5–10.6)	10.0 (8.2–12.2)	2.2 (1.5–3.4)
Streptococcus meningitis§	1.5 (0.9–2.7)	1.7 (1.0–2.7)	1.5 (0.9–2.5)
Other meningitis¶	3.3 (2.2–4.8)	5.3 (4.0–6.9)	3.3 (2.3–4.7)

Incidence rates estimated as the number of hospital admissions per 100 000 person-years at risk.
Analyses included 1 406 547 children.
*ICD-10 code A39.0
†ICD-10 code G00.0
‡ICD-10 code G00.1
§ICD-10 code G00.2
¶ICD-10 codes G00.3-G00.8 and A32.1
CI, confidence intervals. ICD-10, International Classification of Diseases, 10th revision.
