# Supplementary material for: Perinatal factors and hospitalisations for severe childhood infections: a population-based cohort study in Sweden
Source: BMJ Open. 2021 Oct 7;11(10):e054083. doi: 10.1136/bmjopen-2021-054083 (PMC8499334; doi:10.1136/bmjopen-2021-054083)
Supplement: Supplementary data [file bmjopen-2021-054083supp002.pdf]

Appendix B

Table A: Crude and adjusted analyses of the association between perinatal factors and hospitalisations for sepsis and bacterial meningitis in early childhood (28 days until 2 years of age), estimated using multiple imputation

	Sepsis		Bacterial meningitis	
	Crude IRR (95% CI)	Adjusted IRR (95% CI)	Crude IRR (95% CI)	Adjusted IRR (95% CI)
Sex:				
Male	1.07 (0.94–1.22)	1.04 (0.91–1.18)	1.38 (1.14–1.66)	1.36 (1.13–1.64)
Female	1 ref	1 ref	1 ref	1 ref
Gestational age*:				
Extremely preterm	14.83 (10.84–20.30)	9.91 (7.09–13.84)	13.19 (7.23–24.07)	10.85 (5.77–20.41)
Very preterm	5.90 (4.24–8.22)	3.93 (2.74–5.63)	5.06 (2.87–8.94)	4.03 (2.27–7.16)
Moderate preterm	2.50 (2.01–3.10)	2.16 (1.73–2.69)	2.00 (1.45–2.76)	1.84 (1.33–2.55)
Term	1 ref	1 ref	1 ref	1 ref
Post-term	0.80 (0.61–1.04)	0.80 (0.61–1.05)	0.92 (0.62–1.34)	0.88 (0.60–1.30)
SGA:				
Yes	3.47 (2.75–4.37)	2.18 (1.69–2.83)	2.53 (1.61–3.97)	1.63 (1.02–2.62)
No	1 ref	1 ref	1 ref	1 ref
LGA:				
Yes	1.61 (1.17–2.20)	1.61 (1.17–2.21)	1.29 (0.83–2.01)	1.36 (0.87–2.13)
No	1 ref	1 ref	1 ref	1 ref
Congenital malformation†:				
Yes	4.68 (3.95–5.55)	3.96 (3.32–4.71)	2.01 (1.40–2.89)	1.70 (1.18–2.45)
No	1 ref	1 ref	1 ref	1 ref
Maternal age:				
Per year of age	0.99 (0.98–1.00)	0.98 (0.97–1.00)	0.98 (0.97–1.00)	0.99 (0.97–1.01)
Maternal smoking:				
Yes	1.16 (0.94–1.45)	1.00 (0.79–1.26)	1.68 (1.27–2.23)	1.47 (1.08–2.01)
No	1 ref	1 ref	1 ref	1 ref

Pregnancy BMI‡:				
Underweight	0.81 (0.51–1.29)	0.73 (0.46–1.16)	0.90 (0.46–1.74)	0.81 (0.42–1.57)
Normal	1 ref	1 ref	1 ref	1 ref
Overweight	1.12 (0.96–1.32)	1.09 (0.93–1.28)	1.09 (0.85–1.39)	1.05 (0.83–1.35)
Obese	1.24 (1.00–1.53)	1.11 (0.90–1.38)	1.07 (0.79–1.45)	0.98 (0.72–1.34)
Parity:				
Per child	1.06 (1.01–1.12)	1.09 (1.02–1.15)	1.01 (0.92–1.11)	1.00 (0.90–1.10)
Maternal education in years:				
≤9	1.41 (1.15–1.72)	1.15 (0.90–1.46)	1.72 (1.29–2.29)	1.33 (0.95–1.87)
10–12	1.00 (0.85–1.17)	0.91 (0.77–1.09)	1.23 (0.98–1.55)	1.07 (0.83–1.37)
13–14	0.96 (0.77–1.18)	0.93 (0.75–1.16)	1.42 (1.06–1.89)	1.33 (0.99–1.78)
≥15	1 ref	1 ref	1 ref	1 ref
Maternal country of birth:				
Sweden	1 ref	1 ref	1 ref	1 ref
Other Nordic	1.22 (0.79–1.91)	1.20 (0.76–1.87)	1.13 (0.57–2.25)	1.08 (0.54–2.14)
Other Europe and North America	1.43 (1.16–1.76)	1.39 (1.13–1.70)	1.10 (0.78–1.56)	1.08 (0.76–1.53)
Asia	1.20 (0.96–1.49)	1.10 (0.88–1.38)	0.97 (0.68–1.40)	0.97 (0.67–1.39)
Africa	1.20 (0.82–1.75)	1.03 (0.69–1.53)	1.54 (0.98–2.41)	1.57 (1.00–2.46)
Other	0.99 (0.56–1.75)	0.96 (0.54–1.70)	1.93 (0.96–3.87)	1.91 (0.95–3.86)
Year of birth:				
1997–2002	1 ref	1 ref	1 ref	1 ref
2003–2008	1.20 (1.02–1.40)	1.20 (1.02–1.41)	1.23 (1.00–1.52)	1.29 (1.04–1.59)
2009–2013	1.00 (0.85–1.18)	1.00 (0.84–1.19)	0.58 (0.45–0.75)	0.61 (0.47–0.80)
Observations (N):	1 406 547	1 406 547	1 406 547	1 406 547
Clusters (Maternal ID):	891 950	891 950	891 950	891 950

Multiple imputation models included all 1 686 749 children. Adjusted analyses were controlled for sex, gestational age, small for gestational age, large for gestational age, congenital malformation, maternal age, smoking during pregnancy, pregnancy BMI, parity, maternal education level, maternal country of birth and year of birth.

*Gestational age categorised as extremely premature (22–27 weeks), very premature (28–31 weeks), moderate premature (32–36 weeks), term (37–41 weeks) and post-term (≥42 weeks)
†ICD-10 codes: Q00–Q99.
‡BMI categorised as underweight (BMI<18.5 kg/m^2), normal (BMI 18.5–24.9 kg/m^2), overweight (BMI 25.0–29.9 kg/m^2) and obese (BMI≥30.0 kg/m^2).
IRR, incidence rate ratio. CI, confidence intervals. SGA, small for gestational age. LGA, large for gestational age. BMI, body mass index. ICD-10, International Classification of Diseases, 10th revision.
