# Supplementary material for: Perinatal factors and hospitalisations for severe childhood infections: a population-based cohort study in Sweden
Source: BMJ Open. 2021 Oct 7;11(10):e054083. doi: 10.1136/bmjopen-2021-054083 (PMC8499334; doi:10.1136/bmjopen-2021-054083)
Supplement: Author's manuscript [file bmjopen-2021-054083.draft_revisions.pdf]

BMJ Open

BMJ Open is committed to open peer review. As part of this commitment we make the peer review history of every article we publish publicly available.

When an article is published we post the peer reviewers' comments and the authors' responses online. We also post the versions of the paper that were used during peer review. These are the versions that the peer review comments apply to.

The versions of the paper that follow are the versions that were submitted during the peer review process. They are not the versions of record or the final published versions. They should not be cited or distributed as the published version of this manuscript.

BMJ Open is an open access journal and the full, final, typeset and author-corrected version of record of the manuscript is available on our site with no access controls, subscription charges or pay-per-view fees (http://bmjopen.bmj.com).

If you have any questions on BMJ Open's open peer review process please email info.bmjopen@bmj.com

BMJ Open

Perinatal factors and hospitalisations for severe childhood infections: a population-based cohort study in Sweden

Journal:	*BMJ Open*
Manuscript ID	bmjopen-2021-054083
Article Type:	Original research
Date Submitted by the Author:	02-Jun-2021

Complete List of Authors:	Videholm, Samuel; Umea University, Department of Clinical Sciences, Pediatrics<br>Kostenniemi, Urban; Umea University, Department of Clinical Sciences, Pediatrics; Umea University, Department of Clinical Microbiology, Infectious Diseases<br>Lind, Torbjörn ; Umea University, Department of Clinical Sciences, Pediatrics<br>Silfverdal, Sven-Arne; Umea University, Department of Clinical Sciences, Pediatrics

Keywords:	INFECTIOUS DISEASES, Epidemiology < TROPICAL MEDICINE, Paediatric infectious disease & immunisation < PAEDIATRICS, Paediatric A&E and ambulatory care < PAEDIATRICS

SCHOLARONE™ Manuscripts

I, the Submitting Author has the right to grant and does grant on behalf of all authors of the Work (as defined in the below author licence), an exclusive licence and/or a non-exclusive licence for contributions from authors who are: i) UK Crown employees; ii) where BMJ has agreed a CC-BY licence shall apply, and/or iii) in accordance with the terms applicable for US Federal Government officers or employees acting as part of their official duties; on a worldwide, perpetual, irrevocable, royalty-free basis to BMJ Publishing Group Ltd ("BMJ") its licensees and where the relevant Journal is co-owned by BMJ to the co-owners of the Journal, to publish the Work in this journal and any other BMJ products and to exploit all rights, as set out in our licence.

The Submitting Author accepts and understands that any supply made under these terms is made by BMJ to the Submitting Author unless you are acting as an employee on behalf of your employer or a postgraduate student of an affiliated institution which is paying any applicable article publishing charge ("APC") for Open Access articles. Where the Submitting Author wishes to make the Work available on an Open Access basis (and intends to pay the relevant APC), the terms of reuse of such Open Access shall be governed by a Creative Commons licence – details of these licences and which Creative Commons licence will apply to this Work are set out in our licence referred to above.

Other than as permitted in any relevant BMJ Author's Self Archiving Policies, I confirm this Work has not been accepted for publication elsewhere, is not being considered for publication elsewhere and does not duplicate material already published. I confirm all authors consent to publication of this Work and authorise the granting of this licence.

For peer review only

Perinatal factors and hospitalisations for severe childhood infections: a population-based cohort study in Sweden

Samuel Videholm, MD[1], Urban Johansson Kostenniemi, MD, PhD[1,2], Torbjörn Lind, MD, PhD[1], Sven-Arne Silfverdal, MD, PhD, MPH[1]

[1]Department of Clinical Sciences, Pediatrics, Umeå University, Umeå, Sweden

[2]Department of Clinical Microbiology, Infectious Diseases, Umeå University, Umeå, Sweden

Corresponding Author:

Name: Samuel Videholm

E-mail: samuel.videholm@umu.se

Telephone number: +46 907858919

Fax: +46 90123728

Postal address:

Department of Clinical Sciences, Pediatrics,

Umeå University

901 87 Umeå

Sweden

Keywords:

Epidemiology

Infectious Diseases

Paediatric infectious disease & immunization

Paediatric A&E and ambulatory care

Word count: 2510

For peer review only

Abstract

Objective: To examine the association between perinatal factors and hospitalisations for sepsis and meningitis in early childhood (from 28 days until 2 years of age).

Design: A population-based cohort study. The Swedish Medical Birth Register was combined with the National Inpatient Register, the Cause of Death Register, the Total Population Register and the Longitudinal integration database for health insurance and labour market studies.

Setting: Sweden.

Participants: 1 406 547 children born in Sweden between 1997 and 2013.

Main outcome measures: Hospital admissions for sepsis and meningitis recorded between 28 days and 2 years of life.

Results: Gestational age was inversely associated with severe infections i.e. extreme prematurity was strongly associated with an increased risk of sepsis, adjusted incidence rate ratio (aIRR) 10.37 (95% CI 6.78 to 15.86) and meningitis aIRR 6.22 (95% CI 2.28 to 16.94). Presence of congenital malformation was associated with sepsis aIRR 3.89 (95% CI 3.17 to 4.77) and meningitis aIRR 1.69 (95% CI 1.09 to 2.62). Moreover, children born small or large for gestational age were more likely to be hospitalised for sepsis and children exposed to maternal smoking were more likely to be hospitalised for meningitis.

Conclusions: Prematurity and several other perinatal factors were associated with an increased risk of severe infections in young children. Therefore, clinical guidelines for risk assessment of infections in young children should consider perinatal factors.

For peer review only

Strengths and limitations of this study:

The large number of events allowed us to estimate incidence rates of sepsis and meningitis by age in months and year of birth.

The linkage of high-quality health and administrative registers enabled us to examine key birth, pregnancy and sociodemographic characteristics.

Hospitalisations were identified using de-identified hospital discharge data, infections could not be confirmed by laboratory findings.

Information on perinatal factors was missing for 17% of children; nevertheless, results from analyses with imputed data were overall consistent with results from analyses restricted to children with complete data.

For peer review only

BACKGROUND

Sepsis and bacterial meningitis are major causes of morbidity and mortality in young children. After the neonatal period, the global incidence of paediatric sepsis estimated to 1.2 million cases per year and the global incidence of pediatric meningitis is around 280 000 cases per year.[1 2] In high-income countries, sepsis and bacterial meningitis still contribute significantly to the burden of childhood disease.[3-5]

Sepsis and bacterial meningitis are critical illnesses requiring early identification and rapid management, i.e. antibiotic treatment.[6 7] Early identification is challenging; clinical guidelines recommend that clinicians use a combination of symptoms, vital signs and laboratory findings.[7 8] In the neonatal period, clinicians are also recommended to consider perinatal risk factors including gestational age, maternal intraamniotic infection and maternal group B streptococcus colonisation.[9-11] After the neonatal period, children with certain perinatal conditions, e.g. low gestational age and low birth weight are known to be more susceptible to severe infections.[12 13] However, few previous studies have systematically examined the association between perinatal factors and severe infections in young children. Consequently, perinatal risk factors are not regularly considered in clinical guidelines for severe infections after the neonatal period.[7 8 11 14]

Therefore, we examined associations between perinatal factors, i.e. birth, pregnancy and sociodemographic characteristics, and hospitalisations for sepsis and bacterial meningitis in early childhood (from 28 days until 2 years of age).

METHODS

Patient and Public Involvement

This study was conducted without the involvement of patients or members of the public.

Study population

For peer review only

We conducted a population-based cohort study including children born in Sweden between 1997 and 2013, who resided in Sweden at 28 days of age. National health and administrative registers were combined to a dataset using the national registration number, a unique personal identification number assigned to all Swedish residents. The dataset included information from the Medical Birth Register, the National Inpatient Register and the Longitudinal integration database for health, insurance and labour market studies, the Cause of Death Register and the Total Population Register.[15-18] The data were anonymised and linked by the Swedish National Board of Health and Welfare.

Main Exposures

Information on pregnancy and birth characteristics were obtained from the Medical Birth Register. Gestational age was estimated using foetal ultrasound measurements in the early second trimester (around 95%) or information of the last menstrual period and categorised as extremely premature (22–27 weeks), very premature (28–31 weeks), moderate premature (32–36 weeks), term (37–41 weeks) and post-term (≥42 weeks).[19] Sex was categorised as male or female. Small for gestational age (SGA) was defined as a birth weight below the 10th percentile for gestational age at birth.[19] Large for gestational age (LGA) was defined as a birthweight over the 90th percentile for gestational age at birth. Congenital malformation included abnormalities detected during the first 28 days; these malformations were recorded using International Classification of Diseases, 10th revision (ICD-10) codes Q00–Q99.[20] Maternal age was defined as age at delivery. Maternal smoking during pregnancy was self-reported (yes or no) during the first antenatal care visit, normally between 8 and 12 weeks of gestation.[21] Maternal body mass index (BMI) during pregnancy was calculated from weight measured at the first antenatal care visit and self-reported height, and categorised as underweight (<18.5 kg/m^2), normal (18.5–24.9 kg/m^2), overweight (25.0–29.9 kg/m^2) and obese (≥30.0 kg/m^2).[15] Parity was defined as the number of live births including the index child.

For peer review only

Information on maternal education level was retrieved from the Longitudinal integration database for health, insurance and labour market studies and categorised as secondary school or less (≤9 years), upper secondary school (10–12 years), short postsecondary education (13–14 years) and long post-secondary education (≥15 years).

Information on maternal country of birth was obtained from the Total Population Register and categorised as Sweden, Other Nordic, Other Europe and North America, Asia, Africa and Other. The Total Population Register was also used to identify children who migrated and the Cause of Death Register was used to identify children who died.

Outcomes

Information on hospitalisations was retrieved from the National Inpatient Register. We included hospital admissions with a principal diagnosis of sepsis (ICD-10 codes: A39.1-A39.9, A40, A41, A48.3, A02.1 and A32.7) and bacterial meningitis (ICD-10 codes: A39.0, A32.1 and G00) with admission date between 28 days and 2 years of age. Consequently, infections with admission date in the neonatal period were not included. Readmissions within 30 days were excluded.

Statistical analysis

We estimated incidence rates of sepsis and bacterial meningitis as the number of hospital admissions per 100 000 person-years (PY) at risk.

We examined associations between birth (sex, gestational age, SGA, LGA and congenital malformation), pregnancy (smoking during pregnancy, pregnancy BMI, and parity) and sociodemographic (maternal age, maternal education level and maternal country of birth) characteristics, and hospitalisation for sepsis and bacterial meningitis using negative binomial regression models. Crude and adjusted analyses were performed for all variables. In the adjusted analyses, all analyses were adjusted for birth characteristics, pregnancy characteristics, sociodemographic characteristics and time trends (year of birth: 1997–2002, 2003–2008 and 2009–

For peer review only

2013). All models were restricted to observations with complete data on all variables. Log follow up time (in days) was used as offset. Clustering within families was accounted for using generalized estimating equations with robust standard errors.[22] Results were presented as adjusted incidence rate ratios (aIRRs) with 95% confidence intervals (CIs).

In interaction analysis, we examined interactions between sex and prematurity (gestational age <37 weeks), SGA, LGA, congenital malformation and maternal smoking; between prematurity and SGA, LGA, congenital malformation and maternal smoking. Interactions were estimated on an additive scale by calculating the relative excess risk due to interaction (RERI). RERI was calculated using the following formula: RERI = $aIRR_{11} - aIRR_{10} - aIRR_{01} + 1$; 95% CIs were estimated using the delta method. [23 24]

In sensitivity analysis, we imputed missing data using multiple imputation.[25] The predictive model included all variables in the substantive models and both outcomes (hospitalisation for sepsis or bacterial meningitis). Variables were imputed in order of increasing missingness using logistic regression models (categorical variables) and linear regression models (continuous variables). Ten imputed datasets were generated.

All statistical analyses were performed using Stata Statistical Software: Release 14. College Station, Texas, USA.

Ethics

The study was approved by the Regional Ethics Board in Umeå, reference number 2012-265-31M and 2017-399-32M.

RESULTS

Study Population

The Medical Birth Register included 1 686 749 children who resided in Sweden at 28 days of age. We excluded children with missing data (n = 280 202), leaving 1 406 547 children (83% of the original

For peer review only

cohort). All children were followed until 2 years of age or censoring due to death (n = 1417) or emigration (n = 7185). In total, the study included 2 698 039 PY of follow-up time, 1298 hospitalisations for sepsis and 405 hospitalisations for bacterial meningitis.

Table 1 presents background characteristics of the study population. During the study period, 1011 children were hospitalised at least once for sepsis and 382 children were hospitalised at least once for bacterial meningitis. Children hospitalised for severe infections were more likely to have certain characteristics, e.g. premature birth, small for gestational age and low maternal education level.

Incidence rates

Figure 1 shows the incidence rates of sepsis and bacterial meningitis hospitalisations by age in months. The incidence rate of sepsis decreased until the 7th month of life. Thereafter, it remained around 45 hospitalisations per 100 000 PY. The incidence rate of bacterial meningitis decreased steadily during the first year of life. In the second year of life, it remained around 10 hospitalisations per 100 000 PY.

Figure 2 shows the incidence rates of sepsis and bacterial meningitis hospitalisations by year of birth. The incidence rate of bacterial meningitis was around 20 hospitalisations per 100 000 PY in children born 1997–2008. Thereafter, the incidence rate of bacterial meningitis decreased markedly. This was mainly due to a lower incidence rate of pneumococcal meningitis. Online supplementary appendix A includes information on incidence rates for categories of bacterial meningitis grouped by year of birth.

Perinatal factors

Table 2 present the association between perinatal factors and hospitalisations for sepsis and bacterial meningitis. Gestational age was inversely associated with severe infections. In comparison with term children, children born extremely preterm were much more likely to be hospitalised for sepsis aIRR 10.37 (95% CI 6.78 to 15.86) and bacterial meningitis aIRR 6.22 (95% CI 2.28 to 16.94).

For peer review only

Presence of congenital malformation was associated with both sepsis aIRR 3.89 (95% CI 3.17 to 4.77) and bacterial meningitis aIRR 1.69 (95% CI 1.09 to 2.62). An increased risk of sepsis was observed in children born small for gestational age aIRR 2.44 (95% CI 1.83 to 3.25) or large for gestational age aIRR 1.71 (95% CI 1.19 to 2.44). Children born to mothers who smoked during pregnancy were more likely to be hospitalised for bacterial meningitis aIRR 1.46 (95% CI 1.05 to 2.04). Low maternal education (≤9 years) was in comparison with post-secondary education (≥15 years) associated with both sepsis and bacterial meningitis in the unadjusted analyses. Sensitivity analysis showed similar estimates after multiple imputation, except for a stronger association between extreme prematurity and meningitis. Online supplementary appendix B includes results from multiple imputation models.

Table 3 presents results from interaction analyses. An interaction was found between prematurity (gestational age <37 weeks) and congenital malformations on the risk of sepsis RERI 7.75 (95% CI 3.45–12.06); no other interactions were observed.

DISCUSSION

We found that prematurity, in a dose-dependent manner, and congenital malformations were associated with hospitalisations for sepsis and bacterial meningitis in early childhood. Additionally, children born large or small for gestational age were more likely to be hospitalised for sepsis and children exposed to maternal smoking during pregnancy were more likely to be hospitalised for bacterial meningitis. Finally, the incidence of sepsis and bacterial meningitis decreased by the end of the study period.

Incidence rates

The incidence rates of sepsis and bacterial meningitis hospitalisations in our study were similar to those reported in previous studies.[1 3 26] During the study period, the incidence rate of pneumococcal meningitis hospitalisations decreased substantially. This was expected, pneumococcal conjugate vaccines were introduced into the Swedish childhood immunisation program between 2007 and 2009.[27] We have previously reported a decreasing incidence of pneumococcal meningitis after 2009

For peer review only

in Västerbotten county (Sweden).[26] No recent national study has examined the incidence of paediatric meningitis in Sweden.

Perinatal factors

In our study, children born prematurely, small for gestational, large for gestational or with congenital malformations were more likely to be hospitalised for sepsis and/or bacterial meningitis. These children are known to be more susceptible to infections in childhood. In a recent Australian cohort study, low gestational age was associated with an increased risk of infection-related deaths and hospital admissions throughout childhood e.g. gestational age <28 weeks was associated with 2.6 times higher risk of invasive bacterial infections.[12] A large Danish cohort study reported an association between low birth weight and infectious disease-related hospitalisations in the first 15 years of life, e.g. children with a birth weight <1000g were 3.3 times more likely to be hospitalised for sepsis.[13] Another Danish study found that congenital malformations and chromosomal abnormalities were associated with respiratory syncytial virus infections in the first 2 years of life.[28] Overall, these findings are consistent with our results.

We found an association between maternal smoking during pregnancy and bacterial meningitis. This was anticipated from previous research i.e. children exposed to maternal smoking seems to be at risk of meningococcal infections. In a large cohort study from the UK, children exposed to maternal smoking during pregnancy were more likely to be hospitalised for meningococcal meningitis; no association was found for haemophilus meningitis.[29] Moreover, a US study reported that maternal smoking during pregnancy was associated with 2.9 times higher risk of invasive meningococcal disease in children three years and younger.[30] Maternal smoking during pregnancy is a proxy for maternal smoking after birth.[29] Cigarette smoke may increase the risk of bacterial meningitis by decreasing bronchial ciliary activity, decreasing neutrophil function and by predisposing children to viral respiratory infection.[30]

For peer review only

In this study, low maternal education was associated with an increased risk of sepsis and bacterial meningitis. This was expected, numerous studies have shown that social deprivation is associated with an increased risk of disease including paediatric infections. In a Danish cohort study, low maternal education level was associated with 1.3 times higher risk of hospitalisation for infectious diseases in the first 5 years of life.[31] A national cohort study from New Zeeland reported a higher incidence of infection-related hospitalisations in children belonging to economically deprived minorities i.e. the Māori and Pacific peoples.[32] Additionally, a US study reported that low maternal education was associated with a two times higher risk of invasive meningococcal disease in young children.[30] In our study, the association between low maternal education and hospitalisations for sepsis and meningitis decreased and was non-significant in the adjusted analyses. This indicates that the effect of low maternal education was, at least partly, mediated by pregnancy and birth characteristics.

Clinical implications

Pregnancy and birth characteristics are generally considered in clinical guidelines for neonatal infections.[9-11] In contrast, perinatal risk factors are not regularly considered in clinical guidelines for severe infections after the neonatal period, including Swedish and UK guidelines.[7 8 11 14] However, the Swedish guideline recommends that healthcare professionals consider chronic conditions including congenital malformations.[14] We found that prematurity, small or large for gestational age, congenital malformations and maternal smoking during pregnancy were associated with severe infections in young children. Our findings are supported by previous studies.[12 13 28-30] Therefore, we suggest that clinical guidelines for risk assessment of infections after the neonatal period consider perinatal factors as well.

Strengths and limitations

Strengths of this study include the use of high-quality national registers with information on a wide range of perinatal factors. This allowed us to systematically examine associations between perinatal

For peer review only

factors and hospitalisations for severe paediatric infections. However, our study has several limitations. First, infections were identified using de-identified hospital discharge data. Consequently, cases could not be confirmed by laboratory or clinical findings. Secondly, we were unable to examine categories of congenital malformations. This is problematic since susceptibility to infections varies between different congenital conditions.[28] Moreover, the observed interaction between prematurity and congenital malformations on the risk of sepsis may be due to more severe malformations in prematurely born children. Third, all children in Sweden have access to free healthcare and working parents are entitled to paid childcare leave.[33] Consequently, associations between sociodemographic characteristics and severe infections may not be generalised to other settings with less extensive welfare systems. Finally, data was missing for a large proportion (17%) of children. However, results from multiple imputation models were overall consistent with results from the complete case models, indicating no major selection bias.

CONCLUSIONS

In our study, several perinatal factors i.e. prematurity, small for gestational age, large for gestation age, congenital malformations and maternal smoking were associated with an increased risk of sepsis and/or bacterial meningitis in children after the immediate neonatal period up to two years of age. Therefore, we suggest that clinical guidelines for risk assessment of infections in young children consider perinatal factors.

For peer review only

Contributors: SV conceptualised and designed the study together with SAS, carried out analyses and drafted the initial manuscript. UJK contributed to the study design, reviewed and revised the manuscript. TL contributed to the study design, reviewed and revised the manuscript. SAS conceptualised and designed the study together with SV, coordinated and supervised the database, reviewed and revised the manuscript.

Funding: This study was supported by the Unit of Research, Development, and Education, Östersund Hospital, Östersund, Sweden JLL-930202 (to SV); ALF Umeå University, Umeå, Sweden RV-933162 (to SAS).

Competing interests: We have no conflict of interest to declare.

Patient and Public Involvement: This study was conducted without the involvement of patients or members of the public.

Patient consent: Patient informed consent was not needed because the data were collected retrospectively and anonymised prior to analysis.

Ethics approval: The study was approved by the Regional Ethics Board in Umeå, reference number 2012-265-31M and 2017-399-32M.

Acknowledgement: The authors would like to thank Per E. Gustafsson, PhD at the Department of Epidemiology and Global Health, Umeå University, for reviewing and revising the manuscript and Johan Boström, MD at Department of Clinical Sciences, Pediatrics, Umeå University for help in creating the dataset. Håkan Sjöberg, MEc at Statistics Sweden, for help in compiling the dataset.

Data sharing statement: We used deidentified register data obtained from third parties. It includes sensitive information and some access restrictions may apply. Interested researchers need to obtain data directly from the National Board of Health and Welfare in Sweden (socialstyrelsen@socialstyrelsen.se) and Statistics Sweden (scb@scb.se).

For peer review only

Children included in the study were identified in the Medical Birth Register, data on hospitalisations were obtained from the Swedish National Patient Register and data on deaths were obtained from the Cause of Death Register. All of these registers are maintained by the National Board of Health and Welfare in Sweden. Data on maternal education were obtained from the Longitudinal Integration Database for Health Insurance and Labour market Studies and data on migration were obtained from The Total Population Register, both registers are maintained by Statistics Sweden.

For peer review only

REFERENCES

1 Fleischmann-Struzek C, Goldfarb DM, Schlattmann P, Schlapbach LJ, Reinhart K, Kissoon N. The global burden of paediatric and neonatal sepsis: a systematic review. *Lancet Respir Med* 2018;6:223-30.
2 Collaborators GBDM. Global, regional, and national burden of meningitis, 1990-2016: a systematic analysis for the Global Burden of Disease Study 2016. *Lancet Neurol* 2018;17:1061-82.
3 Agyeman PKA, Schlapbach LJ, Giannoni E*, et al.* Epidemiology of blood culture-proven bacterial sepsis in children in Switzerland: a population-based cohort study. *Lancet Child Adolesc Health* 2017;1:124-33.
4 Schlapbach LJ, Straney L, Alexander J*, et al.* Mortality related to invasive infections, sepsis, and septic shock in critically ill children in Australia and New Zealand, 2002-13: a multicentre retrospective cohort study. *Lancet Infect Dis* 2015;15:46-54.
5 Ladhani SN, Collins S, Djennad A*, et al.* Rapid increase in non-vaccine serotypes causing invasive pneumococcal disease in England and Wales, 2000-17: a prospective national observational cohort study. *Lancet Infect Dis* 2018;18:441-51.
6 Weiss SL, Peters MJ, Alhazzani W*, et al.* Surviving sepsis campaign international guidelines for the management of septic shock and sepsis-associated organ dysfunction in children. *Intensive care medicine* 2020;46:10-67.
7 van de Beek D, Cabellos C, Dzupova O*, et al.* ESCMID guideline: diagnosis and treatment of acute bacterial meningitis. *Clinical microbiology and infection : the official publication of the European Society of Clinical Microbiology and Infectious Diseases* 2016;22 Suppl 3:S37-62.
8 National Institute for Health and Care Excellence. Fever in under 5s: assessment and initial management. 2019.
9 Puopolo KM, Benitz WE, Zaoutis TE, Committee On F, Newborn, Committee On Infectious D. Management of Neonates Born at >/=35 0/7 Weeks' Gestation With Suspected or Proven Early-Onset Bacterial Sepsis. *Pediatrics* 2018;142.
10 National Institute for Health and Care Excellence. Neonatal infection (early onset): antibiotics for prevention and treatment. 2012.
11 National Institute for Health and Care Excellence. Sepsis: recognition, diagnosis and early management. 2016.
12 Miller JE, Hammond GC, Strunk T*, et al.* Association of gestational age and growth measures at birth with infection-related admissions to hospital throughout childhood: a population-based, data-linkage study from Western Australia. *Lancet Infect Dis* 2016;16:952-61.
13 Hviid A, Melbye M. The impact of birth weight on infectious disease hospitalization in childhood. *American journal of epidemiology* 2007;165:756-61.
14 The Public Health Agency of Sweden. Tecken på allvarlig infektion hos barn. 2014.
15 The National Board of Health and Welfare in Sweden. The Swedish Medical Birth Register - A summary of content and quality. 2003.
16 Ludvigsson JF, Andersson E, Ekbom A*, et al.* External review and validation of the Swedish national inpatient register. *BMC public health* 2011;11:450.
17 Ludvigsson JF, Svedberg P, Olen O, Bruze G, Neovius M. The longitudinal integrated database for health insurance and labour market studies (LISA) and its use in medical research. *European journal of epidemiology* 2019;34:423-37.
18 Brooke HL, Talback M, Hornblad J*, et al.* The Swedish cause of death register. *European journal of epidemiology* 2017;32:765-73.
19 Ludvigsson JF, Lu D, Hammarstrom L, Cnattingius S, Fang F. Small for gestational age and risk of childhood mortality: A Swedish population study. *PLoS medicine* 2018;15:e1002717.
20 The National Board of Health and Welfare in Sweden. Registration of Congenital Malformations in the Swedish Health Registers. 2004.

For peer review only - http://bmjopen.bmj.com/site/about/guidelines.xhtml

21 Mattsson K, Kallen K, Rignell-Hydbom A*, et al.* Cotinine Validation of Self-Reported Smoking During Pregnancy in the Swedish Medical Birth Register. *Nicotine & tobacco research : official journal of the Society for Research on Nicotine and Tobacco* 2016;18:79-83.
22 Hanley JA, Negassa A, Edwardes MD, Forrester JE. Statistical analysis of correlated data using generalized estimating equations: an orientation. *American journal of epidemiology* 2003;157:364-75.
23 Hosmer DW, Lemeshow S. Confidence interval estimation of interaction. *Epidemiology* 1992;3:452-6.
24 VanderWeele TJ, Knol MJ. A Tutorial on Interaction. *Epidemiologic Methods* 2014;3:33-72.
25 Sterne JA, White IR, Carlin JB*, et al.* Multiple imputation for missing data in epidemiological and clinical research: potential and pitfalls. *Bmj* 2009;338:b2393.
26 Johansson Kostenniemi U, Norman D, Sellin M, Silfverdal SA. Sustained reductions of invasive infectious disease following general infant Haemophilus influenzae type b and pneumococcal vaccination in a Swedish Arctic region. *Acta paediatrica* 2019;108:1871-8.
27 Berglund A, Ekelund M, Fletcher MA, Nyman L. All-cause pneumonia hospitalizations in children <2 years old in sweden, 1998 to 2012: impact of pneumococcal conjugate vaccine introduction. *PloS one* 2014;9:e112211.
28 Kristensen K, Hjuler T, Ravn H, Simoes EA, Stensballe LG. Chronic diseases, chromosomal abnormalities, and congenital malformations as risk factors for respiratory syncytial virus hospitalization: a population-based cohort study. *Clinical infectious diseases : an official publication of the Infectious Diseases Society of America* 2012;54:810-7.
29 Goldacre MJ, Wotton CJ, Maisonneuve JJ. Maternal and perinatal factors associated with subsequent meningococcal, Haemophilus or enteroviral meningitis in children: database study. *Epidemiology and infection* 2014;142:371-8.
30 Yusuf HR, Rochat RW, Baughman WS*, et al.* Maternal cigarette smoking and invasive meningococcal disease: a cohort study among young children in metropolitan Atlanta, 1989-1996. *American journal of public health* 1999;89:712-7.
31 Biering-Sorensen S, Sondergaard G, Vitting Andersen K, Andersen AM, Mortensen LH. Time trends in socio-economic factors and risk of hospitalisation with infectious diseases in pre-school children 1985-2004: a Danish register-based study. *Paediatric and perinatal epidemiology* 2012;26:226-35.
32 Baker MG, Barnard LT, Kvalsvig A*, et al.* Increasing incidence of serious infectious diseases and inequalities in New Zealand: a national epidemiological study. *Lancet* 2012;379:1112-9.
33 Wettergren B, Blennow M, Hjern A, Soder O, Ludvigsson JF. Child Health Systems in Sweden. *The Journal of pediatrics* 2016;177S:S187-S202.

For peer review only

Table 1: Birth, pregnancy and sociodemographic characteristics of the study population

	Included			Excluded*
	Sepsis	Bacterial meningitis	All included	
	n = 1011	n = 382	n = 1 406 547	n = 280 202
Sex:				
Male	55.8 (564)	57.9 (221)	51.4 (722 988)	51.4 (144 079)
Female	44.2 (447)	42.4 (162)	48.6 (683 559)	48.6 (136 117)
Gestation age†:				
Extremely preterm	2.5 (25)	1.0 (4)	0.1 (2012)	0.7 (1927)
Very preterm	2.4 (24)	2.1 (8)	0.4 (5457)	1.6 (4418)
Moderate preterm	8.8 (89)	5.8 (22)	4.0 (56 291)	10.3 (28 979)
Term	80.7 (816)	84.8 (324)	88.3 (1 242 234)	81.4 (227 950)
Post-term	5.6 (57)	6.5 (25)	7.1 (100 553)	5.7 (15 926)
SGA:				
Yes	7.8 (79)	3.7 (14)	2.1 (30 099)	2.1 (5972)
No	92.2 (932)	96.6 (369)	97.9 (1 376 448)	78.7 (220 473)
LGA:				
Yes	5.1 (52)	5.0 (19)	3.7 (51 816)	3.0 (8334)
No	94.9 (959)	95.3 (364)	96.3 (1 354 731)	77.8 (218 111)
Congenital malformation‡:				
Yes	14.1 (143)	6.0 (23)	3.4 (48 209)	3.7 (10 401)
No	85.9 (868)	94.2 (360)	96.6 (1 358 338)	96.3 (269 801)
Maternal age:				
Mean (SD)	30.0 (5.1)	29.7 (5.5)	30.1 (5.1)	30.0 (5.5)
Maternal smoking:				
No smoking	90.6 (916)	86.9 (332)	91.5 (1 286 708)	61.3 (171 790)
Smoking	9.4 (95)	13.4 (51)	8.5 (119 839)	6.9 (19 472)
Pregnancy BMI§:				
Underweight	2.1 (21)	2.4(9)	2.4 (33 836)	1.0 (2895)
Normal	59.8 (605)	60.7 (232)	61.6 (865 874)	20.3 (56 838)
Overweight	25.7 (260)	25.1 (96)	24.8 (348 695)	9.1 (25 600)
Obese	12.4 (125)	12.0 (46)	11.2 (158 142)	4.3 (12 030)
Parity:				
Mean (SD)	1.9 (1.0)	1.8 (1.1)	1.8 (1.0)	2.0 (1.2)
Maternal education in years:				
≤9	14.8 (150)	15.4 (59)	11.6 (162 814)	10.6 (29 682)
10–12	42.0 (425)	42.4 (162)	43.6 (613 370)	36.6 (102 593)
13–14	12.7 (127)	16.0 (61)	13.7 (192 659)	12.6 (35 362)
≥15	30.6 (309)	26.4 (101)	31.1 (437 704)	25.7 (71 907)
Maternal country of birth:				
Sweden	77.3 (782)	80.6 (308)	81.2 (1 142 682)	70.9 (198 735)
Other Nordic	1.8 (18)	1.8 (7)	1.7 (23 403)	2.6 (7173)
Other Europe and North America	8.8 (89)	6.8 (26)	6.2 (87 864)	9.0 (25 278)
Asia	8.6 (87)	6.3 (24)	7.4 (103 858)	10.8 (30 392)

For peer review only

Africa	2.4 (24)	3.4 (13)	2.4 (33 230)	5.2 (14 644)
Other	1.1 (11)	1.3 (5)	1.1 (15 510)	1.3 (3688)
Year of birth:				
1997–2002	27.1 (274)	33.0 (126)	29.5 (41 4783)	40.3 (112 908)
2003–2008	40.6 (410)	46.1 (176)	35.8 (503 841)	37.1 (103 988)
2009–2013	32.3 (327)	21.2 (81)	34.7 (487 923)	22.6 (63 306)

Values are percentages (numbers) unless stated otherwise.

*Children with missing data were excluded (17% of the original cohort). Data on variables were available in 35%–100% of the excluded children.

†Gestational age categorised as extremely premature (22–27 weeks), very premature (28–31 weeks), moderate premature (32–36 weeks), term (37–41 weeks) and post-term (≥42 weeks)

‡ICD-10 codes: Q00–Q99.

§BMI categorised as underweight (BMI<18.5 kg/m^2). normal (BMI 18.5–24.9 kg/m^2). overweight (BMI 25.0–29.9 kg/ m^2) and obese (BMI≥30.0 kg/m^2).

SGA, small for gestational age. LGA, large for gestational age. BMI, body mass index. SD, standard deviation. ICD-10, International Classification of Diseases, 10th revision

For peer review only

Table 2: Crude and adjusted analyses of the association between perinatal factors and hospitalisations for sepsis and bacterial meningitis in early childhood (28 days until 2 years of age)

	Sepsis		Bacterial meningitis	
	Crude IRR (95% CI)	Adjusted IRR (95% CI)	Crude IRR (95% CI)	Adjusted IRR (95% CI)
Sex:				
Male	1.12 (0.97–1.30)	1.09 (0.94–1.26)	1.35 (1.09–1.66)	1.33 (1.08–1.64)
Female	1 ref	1 ref	1 ref	1 ref
Gestational age*:				
Extremely preterm	15.73 (10.49–23.59)	10.37 (6.78–15.86)	7.50 (2.80–20.10)	6.22 (2.28–16.94)
Very preterm	6.44 (4.07–10.18)	4.06 (2.49–6.63)	5.99 (2.89–12.45)	4.95 (2.39–10.24)
Moderate preterm	2.17 (1.71–2.76)	1.85 (1.45–2.37)	1.41 (0.92–2.18)	1.31 (0.84–2.03)
Term	1 ref	1 ref	1 ref	1 ref
Post-term	0.80 (0.59–1.07)	0.80 (0.59–1.08)	0.93 (0.62–1.41)	0.90 (0.59–1.36)
SGA:				
Yes	3.51 (2.72–4.53)	2.44 (1.83–3.25)	1.89 (1.08–3.30)	1.36 (0.78–2.40)
No	1 ref	1 ref	1 ref	1 ref
LGA:				
Yes	1.69 (1.18–2.42)	1.71 (1.19–2.44)	1.43 (0.89–2.31)	1.51 (0.93–2.45)
No	1 ref	1 ref	1 ref	1 ref
Congenital malformation†:				
Yes	4.41 (3.61–5.40)	3.89 (3.17–4.77)	1.86 (1.21–2.88)	1.69 (1.09–2.62)
No	1 ref	1 ref	1 ref	1 ref
Maternal age:				
Per year of age	0.99 (0.98–1.01)	0.99 (0.97–1.00)	0.99 (0.96–1.01)	0.99 (0.97–1.02)
Maternal smoking:				
Yes	1.08 (0.85–1.38)	0.96 (0.75–1.25)	1.65 (1.22–2.24)	1.46 (1.05–2.04)
No	1 ref	1 ref	1 ref	1 ref
Pregnancy BMI‡:				
Underweight	0.84 (0.52–1.37)	0.77 (0.48–1.26)	0.95 (0.49–1.85)	0.88 (0.45–1.71)

For peer review only

Normal	1 ref	1 ref	1 ref	1 ref
Overweight	1.13 (0.95–1.33)	1.10 (0.92–1.30)	1.08 (0.84–1.39)	1.06 (0.82–1.36)
Obese	1.21 (0.96–1.52)	1.11 (0.88–1.39)	1.06 (0.77–1.46)	0.99 (0.72–1.37)
Parity:				
Per child	1.05 (0.98–1.13)	1.08 (1.00–1.16)	0.98 (0.87–1.10)	0.96 (0.85–1.08)
Maternal education in years:				
≤9	1.27 (1.01–1.59)	1.07 (0.83–1.39)	1.70 (1.23–2.36)	1.39 (0.95–2.02)
10–12	0.95 (0.80–1.13)	0.89 (0.74–1.07)	1.23 (0.96–1.59)	1.09 (0.83–1.43)
13–14	0.93 (0.73–1.18)	0.90 (0.71–1.15)	1.46 (1.06–2.02)	1.37 (0.98–1.90)
≥15	1 ref	1 ref	1 ref	1 ref
Maternal country of birth:				
Sweden	1 ref	1 ref	1 ref	1 ref
Other Nordic	1.03 (0.62–1.72)	1.01 (0.61–1.69)	1.23 (0.56–2.70)	1.18 (0.54–2.58)
Other Europe and North America	1.36 (1.07–1.72)	1.33 (1.05–1.70)	1.09 (0.73–1.64)	1.10 (0.73–1.66)
Asia	1.24 (0.96–1.61)	1.17 (0.90–1.53)	0.92 (0.60–1.42)	0.94 (0.61–1.46)
Africa	1.12 (0.65–1.94)	1.00 (0.57–1.75)	1.50 (0.84–2.65)	1.60 (0.91–2.80)
Other	0.88 (0.47–1.62)	0.85 (0.46–1.57)	1.37 (0.54–3.49)	1.38 (0.54–3.52)
Year of birth:				
1997–2002	1 ref	1 ref	1 ref	1 ref
2003–2008	1.26 (1.05–1.50)	1.23 (1.03–1.48)	1.18 (0.93–1.49)	1.23 (0.96–1.57)
2009–2013	1.00 (0.83–1.20)	0.98 (0.80–1.19)	0.54 (0.41–0.72)	0.57 (0.42–0.77)
Observations (N):	1 406 547	1 406 547	1 406 547	1 406 547
Clusters (Maternal ID):	891 950	891 950	891 950	891 950

Analyses excluded children with missing data (n = 280 202), leaving 1 406 547 children. Adjusted analyses were controlled for sex, gestational age, SGA, LGA, congenital malformation, maternal age, smoking during pregnancy, pregnancy BMI, parity, maternal education level, maternal country of birth and year of birth.

For peer review only

*Gestational age categorised as extremely premature (22–27 weeks), very premature (28–31 weeks), moderate premature (32–36 weeks), term (37–41 weeks) and post-term (≥42 weeks)

†ICD-10 codes: Q00–Q99.

‡BMI categorised as underweight (BMI<18.5 kg/m^2), normal (BMI 18.5–24.9 kg/m^2), overweight (BMI 25.0–29.9 kg/m^2) and obese (BMI≥30.0 kg/m^2).

IRR, incidence rate ratio. CI, confidence interval. SGA, small for gestational age. LGA, large for gestational age. BMI, body mass index. ICD-10, International Classification of Diseases, 10th revision.

For peer review only

Table 3: Interactions between sex, prematurity and perinatal factors

	Sepsis	Bacterial meningitis
RERI with sex*:	RERI (95% CI)	RERI (95% CI)
Prematurity*sex	-0.78 (-1.78–0.22)	1.14 (-0.24–2.53)
SGA*sex	-0.45 (-1.87–0.95)	1.83 (-0.08–3.73)
LGA*sex	0.04 (-1.20–1.27)	1.11 (-0.49–2.71)
Congenital malformations*sex	-0.46 (-2.06–1.13)	-0.96 (-2.80–0.88)
Maternal smoking*sex	-0.56 (-1.37–0.25)	0.07 (-0.61–0.75)
RERI with prematurity†:		
SGA*prematurity	1.00 (-1.36–3.36)	0.36 (-2.73–3.45)
LGA*prematurity	-0.97 (-3.01–1.08)	1.58 (-2.76–5.91)
Congenital malformations*prematurity	7.75 (3.45–12.06)	-0.33 (-3.19–2.53)
Maternal smoking*prematurity	-0.51 (-2.12–1.11)	-0.09 (-2.33–2.16)

Analyses excluded children with missing data (n = 280 202), leaving 1 406 547 children. Analyses were controlled for sex, gestational age, SGA, LGA, congenital malformation, maternal age, smoking during pregnancy, pregnancy BMI, parity, maternal education level, maternal country of birth and year of birth. Gestational age was included as a binary variable (0 = gestational age ≥37 weeks, 1 = gestational age <37 weeks); other variables were included as previously described.

*Female is the reference category.

†Gestational age ≥37 weeks is the reference category.

RERI, relative excess risk due to interaction. CI, confidence interval. SGA, small for gestational age. LGA, large for gestational age.

For peer review only

Figure 1. Incidence of sepsis (brown circles) and bacterial meningitis (orange squares) in early childhood, by age in months. Incidence rates calculated as the number of hospital admissions per 100 000 person-years at risk. Vertical lines represent 95% confidence intervals. Analyses included 1 406 547 children.

Figure 2. Incidence of sepsis (brown circles) and bacterial meningitis (orange squares) in early childhood, by year of birth. Incidence rates calculated as the number of hospital admissions per 100 000 person-years at risk. Vertical lines represent 95% confidence intervals. Analyses included 1 406 547 children.

For peer review only

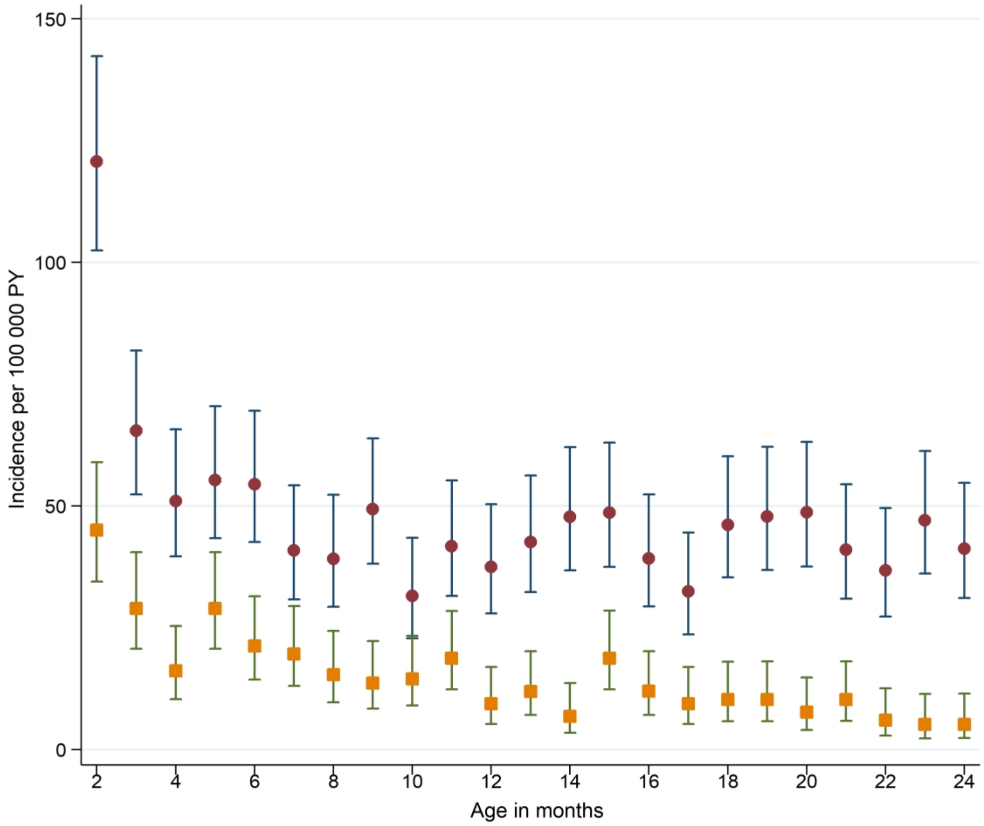

Incidence of sepsis (brown circles) and bacterial meningitis (orange squares) in early childhood, by age in months. Incidence rates calculated as the number of hospital admissions per 100 000 person-years at risk. Vertical lines represent 95% confidence intervals. Analyses included 1 406 547 children.

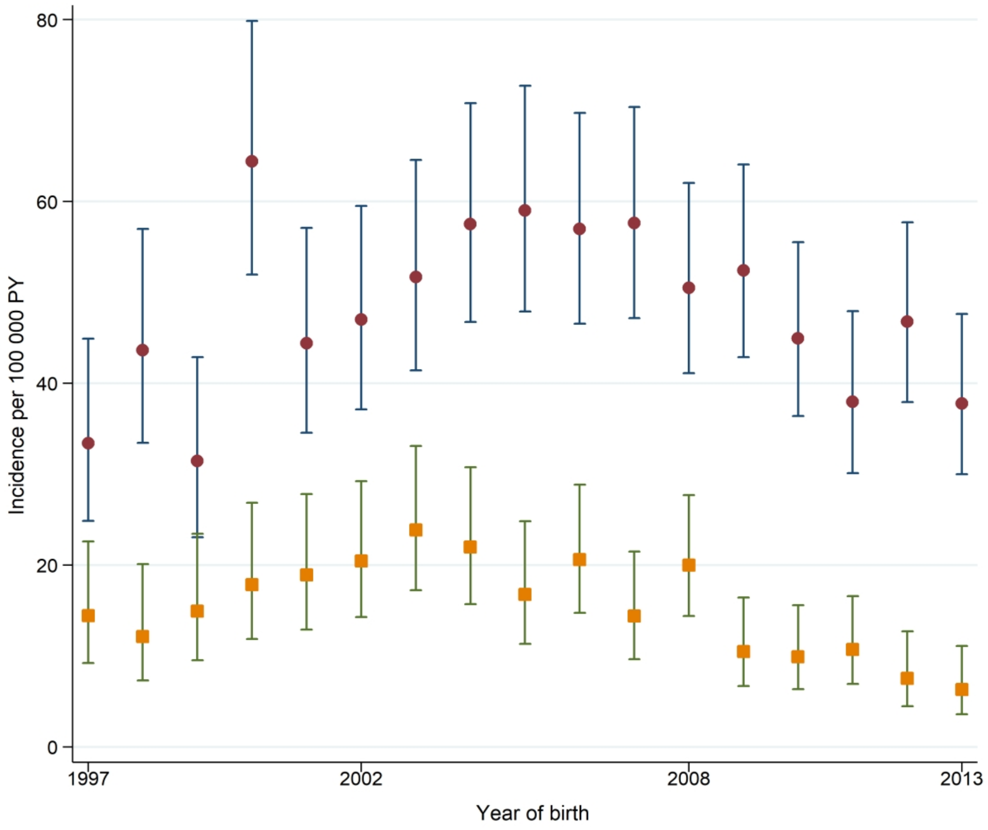

Incidence of sepsis (brown circles) and bacterial meningitis (orange squares) in early childhood, by year of birth. Incidence rates calculated as the number of hospital admissions per 100 000 person-years at risk. Vertical lines represent 95% confidence intervals. Analyses included 1 406 547 children.

Appendix A

Table A: Incidence overall and for categories of bacterial meningitis in early childhood (28 days until 2 years of age), by year of birth

	1997–2002<br>Incidence (95% CI)	2003–2008<br>Incidence (95% CI)	2009–2013<br>Incidence (95% CI)
All	16.6 (14.0–19.7)	19.6 (17.0–22.6)	9.0 (7.2–11.1)
Meningococcal meningitis*	2.5 (1.6–3.9)	1.6 (0.9–2.6)	1.2 (0.7–2.1)
Haemophilus meningitis†	1.0 (0.5–2.0)	1.0 (0.6–1.9)	0.7 (0.4–1.6)
Pneumococcal meningitis‡	8.3 (6.5–10.6)	10.0 (8.2–12.2)	2.2 (1.5–3.4)
Streptococcus meningitis§	1.5 (0.9–2.7)	1.7 (1.0–2.7)	1.5 (0.9–2.5)
Other meningitis¶	3.3 (2.2–4.8)	5.3 (4.0–6.9)	3.3 (2.3–4.7)

Incidence rates estimated as the number of hospital admissions per 100 000 person-years at risk.
Analyses included 1 406 547 children.
*ICD-10 code A39.0
†ICD-10 code G00.0
‡ICD-10 code G00.1
§ICD-10 code G00.2
¶ICD-10 codes G00.3-G00.8 and A32.1
CI, confidence intervals. ICD-10, International Classification of Diseases, 10th revision.

For peer review only

Appendix B

Table A: Crude and adjusted analyses of the association between perinatal factors and hospitalisations for sepsis and bacterial meningitis in early childhood (28 days until 2 years of age), estimated using multiple imputation

	Sepsis		Bacterial meningitis	
	Crude IRR (95% CI)	Adjusted IRR (95% CI)	Crude IRR (95% CI)	Adjusted IRR (95% CI)
Sex:				
Male	1.07 (0.94–1.22)	1.04 (0.91–1.18)	1.38 (1.14–1.66)	1.36 (1.13–1.64)
Female	1 ref	1 ref	1 ref	1 ref
Gestational age*:				
Extremely preterm	14.83 (10.84–20.30)	9.91 (7.09–13.84)	13.19 (7.23–24.07)	10.85 (5.77–20.41)
Very preterm	5.90 (4.24–8.22)	3.93 (2.74–5.63)	5.06 (2.87–8.94)	4.03 (2.27–7.16)
Moderate preterm	2.50 (2.01–3.10)	2.16 (1.73–2.69)	2.00 (1.45–2.76)	1.84 (1.33–2.55)
Term	1 ref	1 ref	1 ref	1 ref
Post-term	0.80 (0.61–1.04)	0.80 (0.61–1.05)	0.92 (0.62–1.34)	0.88 (0.60–1.30)
SGA:				
Yes	3.47 (2.75–4.37)	2.18 (1.69–2.83)	2.53 (1.61–3.97)	1.63 (1.02–2.62)
No	1 ref	1 ref	1 ref	1 ref
LGA:				
Yes	1.61 (1.17–2.20)	1.61 (1.17–2.21)	1.29 (0.83–2.01)	1.36 (0.87–2.13)
No	1 ref	1 ref	1 ref	1 ref
Congenital malformation†:				
Yes	4.68 (3.95–5.55)	3.96 (3.32–4.71)	2.01 (1.40–2.89)	1.70 (1.18–2.45)
No	1 ref	1 ref	1 ref	1 ref
Maternal age:				
Per year of age	0.99 (0.98–1.00)	0.98 (0.97–1.00)	0.98 (0.97–1.00)	0.99 (0.97–1.01)
Maternal smoking:				
Yes	1.16 (0.94–1.45)	1.00 (0.79–1.26)	1.68 (1.27–2.23)	1.47 (1.08–2.01)
No	1 ref	1 ref	1 ref	1 ref
Pregnancy BMI‡:				

For peer review only

Underweight	0.81 (0.51–1.29)	0.73 (0.46–1.16)	0.90 (0.46–1.74)	0.81 (0.42–1.57)
Normal	1 ref	1 ref	1 ref	1 ref
Overweight	1.12 (0.96–1.32)	1.09 (0.93–1.28)	1.09 (0.85–1.39)	1.05 (0.83–1.35)
Obese	1.24 (1.00–1.53)	1.11 (0.90–1.38)	1.07 (0.79–1.45)	0.98 (0.72–1.34)
Parity:				
Per child	1.06 (1.01–1.12)	1.09 (1.02–1.15)	1.01 (0.92–1.11)	1.00 (0.90–1.10)
Maternal education in years:				
≤9	1.41 (1.15–1.72)	1.15 (0.90–1.46)	1.72 (1.29–2.29)	1.33 (0.95–1.87)
10–12	1.00 (0.85–1.17)	0.91 (0.77–1.09)	1.23 (0.98–1.55)	1.07 (0.83–1.37)
13–14	0.96 (0.77–1.18)	0.93 (0.75–1.16)	1.42 (1.06–1.89)	1.33 (0.99–1.78)
≥15	1 ref	1 ref	1 ref	1 ref
Maternal country of birth:				
Sweden	1 ref	1 ref	1 ref	1 ref
Other Nordic	1.22 (0.79–1.91)	1.20 (0.76–1.87)	1.13 (0.57–2.25)	1.08 (0.54–2.14)
Other Europe and North America	1.43 (1.16–1.76)	1.39 (1.13–1.70)	1.10 (0.78–1.56)	1.08 (0.76–1.53)
Asia	1.20 (0.96–1.49)	1.10 (0.88–1.38)	0.97 (0.68–1.40)	0.97 (0.67–1.39)
Africa	1.20 (0.82–1.75)	1.03 (0.69–1.53)	1.54 (0.98–2.41)	1.57 (1.00–2.46)
Other	0.99 (0.56–1.75)	0.96 (0.54–1.70)	1.93 (0.96–3.87)	1.91 (0.95–3.86)
Year of birth:				
1997–2002	1 ref	1 ref	1 ref	1 ref
2003–2008	1.20 (1.02–1.40)	1.20 (1.02–1.41)	1.23 (1.00–1.52)	1.29 (1.04–1.59)
2009–2013	1.00 (0.85–1.18)	1.00 (0.84–1.19)	0.58 (0.45–0.75)	0.61 (0.47–0.80)
Observations (N):	1 406 547	1 406 547	1 406 547	1 406 547
Clusters (Maternal ID):	891 950	891 950	891 950	891 950

Multiple imputation models included all 1 686 749 children. Adjusted analyses were controlled for sex, gestational age, small for gestational age, large for gestational age, congenital malformation, maternal age, smoking during pregnancy, pregnancy BMI, parity, maternal education level, maternal country of birth and year of birth.

For peer review only

*Gestational age categorised as extremely premature (22–27 weeks), very premature (28–31 weeks), moderate premature (32–36 weeks), term (37–41 weeks) and post-term (≥42 weeks)
†ICD-10 codes: Q00–Q99.
‡BMI categorised as underweight (BMI<18.5 kg/m^2), normal (BMI 18.5–24.9 kg/m^2), overweight (BMI 25.0–29.9 kg/m^2) and obese (BMI≥30.0 kg/m^2).
IRR, incidence rate ratio. CI, confidence intervals. SGA, small for gestational age. LGA, large for gestational age. BMI, body mass index. ICD-10, International Classification of Diseases, 10th revision.

For peer review only

STROBE Statement—Checklist of items that should be included in reports of ***cohort studies***

	Item No	Recommendation	Page No
**Title and abstract**	1	(*a*) Indicate the study's design with a commonly used term in the title or the abstract	1
		(*b*) Provide in the abstract an informative and balanced summary of what was done and what was found	2
**Introduction**			
Background/rationale	2	Explain the scientific background and rationale for the investigation being reported	4
Objectives	3	State specific objectives, including any prespecified hypotheses	4
**Methods**			
Study design	4	Present key elements of study design early in the paper	5
Setting	5	Describe the setting, locations, and relevant dates, including periods of recruitment, exposure, follow-up, and data collection	5
Participants	6	(*a*) Give the eligibility criteria, and the sources and methods of selection of participants. Describe methods of follow-up	5
		(*b*) For matched studies, give matching criteria and number of exposed and unexposed	
Variables	7	Clearly define all outcomes, exposures, predictors, potential confounders, and effect modifiers. Give diagnostic criteria, if applicable	5-6
Data sources/ measurement	8*	For each variable of interest, give sources of data and details of methods of assessment (measurement). Describe comparability of assessment methods if there is more than one group	5-6
Bias	9	Describe any efforts to address potential sources of bias	7
Study size	10	Explain how the study size was arrived at	-
Quantitative variables	11	Explain how quantitative variables were handled in the analyses. If applicable, describe which groupings were chosen and why	5-6
Statistical methods	12	(*a*) Describe all statistical methods, including those used to control for confounding	6-7
		(*b*) Describe any methods used to examine subgroups and interactions	
		(*c*) Explain how missing data were addressed	
		(*d*) If applicable, explain how loss to follow-up was addressed	
		(*e*) Describe any sensitivity analyses	
**Results**			
Participants	13*	(a) Report numbers of individuals at each stage of study—eg numbers potentially eligible, examined for eligibility, confirmed eligible, included in the study, completing follow-up, and analysed	7-8
		(b) Give reasons for non-participation at each stage	
		(c) Consider use of a flow diagram	
Descriptive data	14*	(a) Give characteristics of study participants (eg demographic, clinical, social) and information on exposures and potential confounders	8 + Table 1
		(b) Indicate number of participants with missing data for each variable of interest	Table 1
		(c) Summarise follow-up time (eg, average and total amount)	8
Outcome data	15*	Report numbers of outcome events or summary measures over time	8

For peer review only

For peer review only - http://bmjopen.bmj.com/site/about/guidelines.xhtml

Main results	16	(*a*) Give unadjusted estimates and, if applicable, confounder-adjusted estimates and their precision (eg, 95% confidence interval). Make clear which confounders were adjusted for and why they were included<br>(*b*) Report category boundaries when continuous variables were categorized<br>(*c*) If relevant, consider translating estimates of relative risk into absolute risk for a meaningful time period	8-9<br>Table2
Other analyses	17	Report other analyses done—eg analyses of subgroups and interactions, and sensitivity analyses	Table3 Appendix B
**Discussion**			
Key results	18	Summarise key results with reference to study objectives	9
Limitations	19	Discuss limitations of the study, taking into account sources of potential bias or imprecision. Discuss both direction and magnitude of any potential bias	11-12
Interpretation	20	Give a cautious overall interpretation of results considering objectives, limitations, multiplicity of analyses, results from similar studies, and other relevant evidence	9-11
Generalisability	21	Discuss the generalisability (external validity) of the study results	12
**Other information**			
Funding	22	Give the source of funding and the role of the funders for the present study and, if applicable, for the original study on which the present article is based	13

*Give information separately for exposed and unexposed groups.

Note: An Explanation and Elaboration article discusses each checklist item and gives methodological background and published examples of transparent reporting. The STROBE checklist is best used in conjunction with this article (freely available on the Web sites of PLoS Medicine at http://www.plosmedicine.org/, Annals of Internal Medicine at http://www.annals.org/, and Epidemiology at http://www.epidem.com/). Information on the STROBE Initiative is available at http://www.strobe-statement.org.

For peer review only

BMJ Open

Perinatal factors and hospitalisations for severe childhood infections: a population-based cohort study in Sweden

Journal:	*BMJ Open*
Manuscript ID	bmjopen-2021-054083.R1
Article Type:	Original research
Date Submitted by the Author:	27-Jul-2021

Complete List of Authors:	Videholm, Samuel; Umea University, Department of Clinical Sciences, Pediatrics<br>Kostenniemi, Urban; Umea Universitet, Department of Clinical Sciences, Pediatrics; Umea University, Department of Clinical Microbiology, Infectious Diseases<br>Lind, Torbjörn ; Umea University, Department of Clinical Sciences, Pediatrics<br>Silfverdal, Sven-Arne; Umea University, Department of Clinical Sciences, Pediatrics

**Primary Subject Heading**:	Epidemiology
Secondary Subject Heading:	Infectious diseases
Keywords:	INFECTIOUS DISEASES, Epidemiology < TROPICAL MEDICINE, Paediatric infectious disease & immunisation < PAEDIATRICS, Paediatric A&E and ambulatory care < PAEDIATRICS

SCHOLARONE™
Manuscripts

I, the Submitting Author has the right to grant and does grant on behalf of all authors of the Work (as defined in the below author licence), an exclusive licence and/or a non-exclusive licence for contributions from authors who are: i) UK Crown employees; ii) where BMJ has agreed a CC-BY licence shall apply, and/or iii) in accordance with the terms applicable for US Federal Government officers or employees acting as part of their official duties; on a worldwide, perpetual, irrevocable, royalty-free basis to BMJ Publishing Group Ltd ("BMJ") its licensees and where the relevant Journal is co-owned by BMJ to the co-owners of the Journal, to publish the Work in this journal and any other BMJ products and to exploit all rights, as set out in our licence.

The Submitting Author accepts and understands that any supply made under these terms is made by BMJ to the Submitting Author unless you are acting as an employee on behalf of your employer or a postgraduate student of an affiliated institution which is paying any applicable article publishing charge ("APC") for Open Access articles. Where the Submitting Author wishes to make the Work available on an Open Access basis (and intends to pay the relevant APC), the terms of reuse of such Open Access shall be governed by a Creative Commons licence – details of these licences and which Creative Commons licence will apply to this Work are set out in our licence referred to above.

Other than as permitted in any relevant BMJ Author's Self Archiving Policies, I confirm this Work has not been accepted for publication elsewhere, is not being considered for publication elsewhere and does not duplicate material already published. I confirm all authors consent to publication of this Work and authorise the granting of this licence.

For peer review only

Perinatal factors and hospitalisations for severe childhood infections: a population-based cohort study in Sweden

Samuel Videholm, MD[1], Urban Johansson Kostenniemi, MD, PhD[1,2], Torbjörn Lind, MD, PhD[1], Sven-Arne Silfverdal, MD, PhD, MPH[1]

[1]Department of Clinical Sciences, Pediatrics, Umeå University, Umeå, Sweden

[2]Department of Clinical Microbiology, Infectious Diseases, Umeå University, Umeå, Sweden

Corresponding Author:

Name: Samuel Videholm

E-mail: samuel.videholm@umu.se

Telephone number: +46 907858919

Fax: +46 90123728

Postal address:

Department of Clinical Sciences, Pediatrics,

Umeå University

901 87 Umeå

Sweden

Keywords:

Epidemiology

Infectious Diseases

Paediatric infectious disease & immunization

Paediatric A&E and ambulatory care

Word count: 2812

For peer review only

Abstract

Objective: To examine the association between perinatal factors and hospitalisations for sepsis and bacterial meningitis in early childhood (from 28 days until 2 years of age).

Design: A population-based cohort study. The Swedish Medical Birth Register was combined with the National Inpatient Register, the Cause of Death Register, the Total Population Register and the Longitudinal integration database for health insurance and labour market studies. Associations between perinatal factors and hospitalisations were examined using negative binomial regression models.

Setting: Sweden.

Participants: 1 406 547 children born in Sweden between 1997 and 2013.

Main outcome measures: Hospital admissions for sepsis and bacterial meningitis recorded between 28 days and 2 years of life.

Results: Gestational age was inversely associated with severe infections i.e. extreme prematurity was strongly associated with an increased risk of sepsis, adjusted incidence rate ratio (aIRR) 10.37 (95% CI 6.78 to 15.86) and meningitis aIRR 6.22 (95% CI 2.28 to 16.94). Presence of congenital malformation was associated with sepsis aIRR 3.89 (95% CI 3.17 to 4.77) and meningitis aIRR 1.69 (95% CI 1.09 to 2.62). Moreover, children born small or large for gestational age were more likely to be hospitalised for sepsis and children exposed to maternal smoking were more likely to be hospitalised for meningitis.

Conclusions: Prematurity and several other perinatal factors were associated with an increased risk of severe infections in young children. Therefore, clinical guidelines for risk assessment of infections in young children should consider perinatal factors.

For peer review only

Strengths and limitations of this study:

The large number of events allowed us to estimate incidence rates of sepsis and bacterial meningitis by age in months and year of birth.

The linkage of high-quality health and administrative registers enabled us to examine key birth, pregnancy and sociodemographic characteristics.

Hospitalisations were identified using de-identified hospital discharge data; infections could not be confirmed by laboratory findings.

Information on perinatal factors was missing for 17% of children; nevertheless, results from analyses with imputed data were overall consistent with results from analyses restricted to children with complete data.

For peer review only

BACKGROUND

Sepsis and bacterial meningitis are major causes of morbidity and mortality in young children. After the neonatal period (from birth until 28 days), the global incidence of paediatric sepsis estimated to 1.2 million cases per year in 2013 and the global incidence of pediatric meningitis is around 280 000 cases per year in 2016.[1 2] In high-income countries, sepsis and bacterial meningitis still contribute significantly to the burden of childhood disease.[3-5]

Sepsis and bacterial meningitis are critical illnesses requiring early identification and rapid management, i.e. antibiotic treatment.[6 7] Early identification is challenging; clinical guidelines recommend that clinicians use a combination of symptoms, vital signs and laboratory findings.[7 8] In the neonatal period, clinicians are also recommended to consider perinatal risk factors including gestational age, maternal intraamniotic infection and maternal group B streptococcus colonisation.[9-11] After the neonatal period, children with certain perinatal conditions, e.g. low gestational age and low birth weight are known to be more susceptible to severe infections.[12 13] However, perinatal risk factors are not regularly considered in clinical guidelines for severe infections after the neonatal period.[7 8 11 14]

Therefore, we examined associations between perinatal factors, i.e. birth, pregnancy and sociodemographic characteristics, and hospitalisations for sepsis and bacterial meningitis in early childhood (from 28 days until 2 years of age).

METHODS

Patient and Public Involvement

This study used retrospectively collected register data. Therefore, patients or members of the public were not involved in the design or conduct of this study.

Study population

For peer review only - http://bmjopen.bmj.com/site/about/guidelines.xhtml

We conducted a population-based cohort study including children born in Sweden between 1997 and 2013, who resided in Sweden at 28 days of age. National health and administrative registers were combined to a dataset using the national registration number, a unique personal identification number assigned to all Swedish residents. The dataset included information from the Medical Birth Register which covers over 98-99% of all births and contains information on prenatal, delivery and neonatal care, the National Inpatient Register which holds information on 99% of all inpatient hospital admissions, the Longitudinal integration database for health, insurance and labour market studies which contains socio-economic data, the Cause of Death Register which covers over 97% of all deaths and the Total Population Register.[15-18] The data were anonymised and linked by the Swedish National Board of Health and Welfare.

Main Exposures

Information on pregnancy and birth characteristics were obtained from the Medical Birth Register. Gestational age was estimated using foetal ultrasound measurements in the early second trimester (performed in around 95% of pregnancies) or information of the last menstrual period and categorised as extremely premature (22–27 weeks), very premature (28–31 weeks), moderate premature (32–36 weeks), term (37–41 weeks) and post-term (≥42 weeks).[19] Sex was categorised as male or female. Small for gestational age (SGA) was defined as a birth weight below the 10th percentile for gestational age at birth.[19] Large for gestational age (LGA) was defined as a birthweight over the 90th percentile for gestational age at birth. Congenital malformation included abnormalities detected during the first 28 days; these malformations were recorded using International Classification of Diseases, 10th revision (ICD-10) codes Q00–Q99.[20] Maternal age was defined as age at delivery. Maternal smoking during pregnancy was self-reported (yes or no) during the first antenatal care visit, normally between 8 and 12 weeks of gestation.[21] Maternal body mass index (BMI) during pregnancy was calculated from weight measured at the first antenatal care visit and self-reported height, and categorised as underweight (<18.5 kg/m^2), normal (18.5–24.9 kg/m^2),

For peer review only

overweight (25.0–29.9 kg/m^2) and obese (≥30.0 kg/m^2).[15] Parity was defined as the number of live births including the index child.

Information on maternal education level was retrieved from the Longitudinal integration database for health, insurance and labour market studies and categorised as secondary school or less (≤9 years), upper secondary school (10–12 years), short postsecondary education (13–14 years) and long post-secondary education (≥15 years).

Information on maternal country of birth was obtained from the Total Population Register and categorised as Sweden, Other Nordic, Other Europe and North America, Asia, Africa and Other. The Total Population Register was also used to identify children who migrated and the Cause of Death Register was used to identify children who died.

Outcomes

The two main outcomes were number of hospitalisations for sepsis and number of hospitalisations for bacterial meningitis. Information on hospital admissions with a principal diagnosis of sepsis (ICD-10 codes: A39.1-A39.9, A40, A41, A48.3, A02.1 and A32.7) and bacterial meningitis (ICD-10 codes: A39.0, A32.1 and G00) were retrieved from the National Inpatient Register. Only hospitalisations with admission dates between 28 days and 2 years of age were included. Consequently, infections with admission date in the neonatal period were not included. Readmissions within 30 days were excluded.

Statistical analysis

We estimated incidence rates of sepsis and bacterial meningitis as the number of hospital admissions per 100 000 person-years (PY) at risk.

We examined associations between birth (sex, gestational age, SGA, LGA and congenital malformation), pregnancy (smoking during pregnancy, pregnancy BMI, and parity) and sociodemographic (maternal age, maternal education level and maternal country of birth)

For peer review only

characteristics, and hospitalisation for sepsis and bacterial meningitis using negative binomial regression models. Crude and adjusted analyses were performed for all variables. In the adjusted analyses, all analyses were adjusted for birth characteristics, pregnancy characteristics, sociodemographic characteristics and time trends (year of birth: 1997–2002, 2003–2008 and 2009–2013). All models were restricted to observations with complete data on all variables. Log follow up time (in days) was used as offset. Clustering within families was accounted for using generalized estimating equations with robust standard errors.[22] Results were presented as adjusted incidence rate ratios (aIRRs) with 95% confidence intervals (CIs).

In interaction analysis, we examined interactions between sex and prematurity (gestational age <37 weeks), SGA, LGA, congenital malformation and maternal smoking; between prematurity and SGA, LGA, congenital malformation and maternal smoking. Interactions were estimated on an additive scale by calculating the relative excess risk due to interaction (RERI). RERI was calculated using the following formula: RERI = $aIRR_{11} – aIRR_{10} – aIRR_{01} + 1$; 95% CIs were estimated using the delta method. [23,24]

In sensitivity analysis, we imputed missing data using multiple imputation.[25] The predictive model included all variables in the substantive models and both outcomes (hospitalisation for sepsis or bacterial meningitis). Variables were imputed in order of increasing missingness using logistic regression models (categorical variables) and linear regression models (continuous variables). Ten imputed datasets were generated.

All statistical analyses were performed using Stata Statistical Software: Release 14. College Station, Texas, USA.

Ethics

The study was approved by the Regional Ethics Board in Umeå, reference number 2012-265-31M and 2017-399-32M.

For peer review only

RESULTS

Study Population

The Medical Birth Register included 1 690 100 live births between 1997 and 2013. Children who died (n = 3193) or emigrated (n = 158) in the neonatal period were removed, leaving 1 686 749 children who resided in Sweden at 28 days of age (the target population). We excluded children with missing data (n = 280 202), leaving 1 406 547 children (figure 1). All children were followed until 2 years of age or censoring due to death (n = 1417) or emigration (n = 7185). In total, the study included 2 698 039 PY of follow-up time, 1298 hospitalisations for sepsis and 405 hospitalisations for bacterial meningitis.

Table 1 presents background characteristics of the study population. During the study period, 1011 children were hospitalised at least once for sepsis, 382 children were hospitalised at least once for bacterial meningitis and 18 children were hospitalised at least once for both sepsis and bacterial meningitis. Children hospitalised for severe infections were more likely to have certain characteristics, e.g. premature birth, small for gestational age and low maternal education level.

Incidence rates

Figure 2 shows the incidence rates of sepsis and bacterial meningitis hospitalisations by age in months. The incidence rate of sepsis decreased until the 3rd month of life. Thereafter, it remained around 45 hospitalisations per 100 000 PY. The incidence rate of bacterial meningitis decreased until the 3rd month of life, thereafter it was around 20 hospitalisations per 100 000 PY in the first year of life and around 10 hospitalisations per 100 000 PY in the second year of life.

Figure 3 shows the incidence rates of sepsis and bacterial meningitis hospitalisations by year of birth. The incidence rate of bacterial meningitis was around 20 hospitalisations per 100 000 PY in children born 1997–2008. Thereafter, the incidence rate of bacterial meningitis decreased markedly. This was mainly due to a lower incidence rate of pneumococcal meningitis. Online supplementary appendix A

For peer review only

includes information on incidence rates for categories of bacterial meningitis grouped by year of birth.

Perinatal factors

Table 2 present the association between perinatal factors and hospitalisations for sepsis and bacterial meningitis. Gestational age was inversely associated with severe infections. In comparison with term children, children born extremely preterm were much more likely to be hospitalised for sepsis aIRR 10.37 (95% CI 6.78 to 15.86) and bacterial meningitis aIRR 6.22 (95% CI 2.28 to 16.94). Presence of congenital malformation was associated with both sepsis aIRR 3.89 (95% CI 3.17 to 4.77) and bacterial meningitis aIRR 1.69 (95% CI 1.09 to 2.62). An increased risk of sepsis was observed in children born small for gestational age aIRR 2.44 (95% CI 1.83 to 3.25) or large for gestational age aIRR 1.71 (95% CI 1.19 to 2.44). Children born to mothers who smoked during pregnancy were more likely to be hospitalised for bacterial meningitis aIRR 1.46 (95% CI 1.05 to 2.04). Low maternal education (≤9 years) was in comparison with post-secondary education (≥15 years) associated with both sepsis and bacterial meningitis in the unadjusted analyses. Sensitivity analysis showed similar estimates after multiple imputation, except for a stronger association between extreme prematurity and meningitis. Online supplementary appendix B includes results from multiple imputation models.

Table 3 presents results from interaction analyses. An additive interaction was found between prematurity (gestational age <37 weeks) and congenital malformations on the risk of sepsis RERI 7.75 (95% CI 3.45–12.06); no other additive interactions were observed.

DISCUSSION

We found that prematurity, in a dose-dependent manner, and congenital malformations were associated with hospitalisations for sepsis and bacterial meningitis in early childhood. Additionally, children born large or small for gestational age were more likely to be hospitalised for sepsis and children exposed to maternal smoking during pregnancy were more likely to be hospitalised for

bacterial meningitis. Finally, the incidence of sepsis and bacterial meningitis decreased by the end of the study period.

Incidence rates

The incidence rates of sepsis and bacterial meningitis hospitalisations in our study were similar to those reported in previous studies.[1 3 26] During the study period, the incidence rate of pneumococcal meningitis hospitalisations decreased substantially. This was expected, pneumococcal conjugate vaccines were introduced into the Swedish childhood immunisation program between 2007 and 2009.[27] We have previously reported a decreasing incidence of pneumococcal meningitis after 2009 in Västerbotten county (Sweden).[26] No recent national study has examined the incidence of paediatric meningitis in Sweden.

Perinatal factors

In our study, children born prematurely, small for gestational age, with congenital malformations or with male sex were more likely to be hospitalised for sepsis and/or bacterial meningitis. These children are known to be more susceptible to infections in childhood. In a recent Australian cohort study, low gestational age was associated with an increased risk of infection-related deaths and hospital admissions throughout childhood e.g. gestational age <28 weeks was associated with 2.6 times higher risk of invasive bacterial infections.[12] A large Danish cohort study reported an association between low birth weight and infectious disease-related hospitalisations in the first 15 years of life, e.g. children with a birth weight <1000g were 3.3 times more likely to be hospitalised for sepsis.[13] Another Danish study found that congenital malformations and chromosomal abnormalities were associated with respiratory syncytial virus infections in the first 2 years of life.[28] Finally, previous studies have shown that male sex is associated with a large number of childhood infections, including sepsis and meningitis.[3 29 30] Differences in sex hormones has been suggested as one possible explanation, testosterone has an overall suppressive effect on the immune system whereas estrogen

For peer review only

promotes Th1 cellular immune responses and humoral immunity.[29] Overall, these findings are consistent with our results.

In this study, children born large for gestational age were more likely to be hospitalised for sepsis and bacterial meningitis. One explanation could be a higher incidence of maternal diabetes in children born large for gestational age. Both maternal pre-existing diabetes and gestational diabetes are associated with an increased risk of childhood infections.[31] Similar to our results, a national cohort study reported an increased risk for sepsis in children with a birth weight >4500g; however, in contrast to our results, the risk of bacterial meningitis was not increased in heavy children.[13] Therefore, our findings must be interpreted with caution.

We found an association between maternal smoking during pregnancy and bacterial meningitis. This was anticipated from previous research i.e. children exposed to maternal smoking seems to be at risk of meningococcal infections. In a large cohort study from the UK, children exposed to maternal smoking during pregnancy were more likely to be hospitalised for meningococcal meningitis; no association was found for haemophilus meningitis.[32] Moreover, a US study reported that maternal smoking during pregnancy was associated with 2.9 times higher risk of invasive meningococcal disease in children three years and younger.[30] Maternal smoking during pregnancy is a proxy for maternal smoking after birth.[32] Cigarette smoke may increase the risk of bacterial meningitis by decreasing bronchial ciliary activity, decreasing neutrophil function and by predisposing children to viral respiratory infection.[30]

In our study, low maternal education was associated with an increased risk of sepsis and bacterial meningitis in the crude analyses. This was expected, numerous studies have shown that social deprivation is associated with an increased risk of disease including paediatric infections. In a Danish cohort study, low maternal education level was associated with 1.3 times higher risk of hospitalisation for infectious diseases in the first 5 years of life.[33] A national cohort study from New Zealand reported a higher incidence of infection-related hospitalisations in children belonging to

For peer review only

economically deprived minorities i.e. the Māori and Pacific peoples.[34] Additionally, a US study reported that low maternal education was associated with a two times higher risk of invasive meningococcal disease in young children.[30] In our study, the association between low maternal education and hospitalisations for sepsis and meningitis decreased and was non-significant in the adjusted analyses. This indicates that the effect of low maternal education was, at least partly, mediated by pregnancy and birth characteristics.

Clinical implications

Pregnancy and birth characteristics are generally considered in clinical guidelines for neonatal infections.[9-11] In contrast, perinatal risk factors are not regularly considered in clinical guidelines for severe infections after the neonatal period, including Swedish and UK guidelines.[7 8 11 14] However, the Swedish guideline recommends that healthcare professionals consider chronic conditions including congenital malformations.[14] We found that prematurity, small or large for gestational age, congenital malformations and maternal smoking during pregnancy were associated with severe infections in young children. Our findings are supported by previous studies.[12 13 28 30 32] Therefore, we suggest that clinical guidelines for risk assessment of infections after the neonatal period consider perinatal factors as well.

Strengths and limitations

Strengths of this study include the use of high-quality national registers with information on a wide range of perinatal factors. This allowed us to systematically examine associations between perinatal factors and hospitalisations for severe paediatric infections. However, our study has several limitations. First, infections were identified using de-identified hospital discharge data. Consequently, cases could not be confirmed by laboratory findings or by other information obtained from medical records. Secondly, we were unable to examine categories of congenital malformations. This is problematic since susceptibility to infections varies between different congenital conditions.[28] Moreover, the observed interaction between prematurity and congenital malformations on the risk

For peer review only

of sepsis may be due to more severe malformations in prematurely born children. Third, all children in Sweden have access to free healthcare and working parents are entitled to paid childcare leave.[35] Consequently, associations between sociodemographic characteristics and severe infections may not be generalised to other settings with less extensive welfare systems. Fourth, our study lacks information on postnatal factors associated with infectious diseases e.g. acquired chronic diseases, vaccination status and breastfeeding. Finally, data was missing for a large proportion (17%) of children. However, results from multiple imputation models were overall consistent with results from the complete case models, indicating no major selection bias.

CONCLUSIONS

In our study, several perinatal factors i.e. prematurity, small for gestational age, large for gestation age, congenital malformations and maternal smoking were associated with an increased risk of sepsis and/or bacterial meningitis in children after the immediate neonatal period up to two years of age. Therefore, we suggest that clinical guidelines for risk assessment of infections in young children consider perinatal factors.

For peer review only

Contributors: SV conceptualised and designed the study together with SAS, carried out analyses and drafted the initial manuscript. UJK contributed to the study design, reviewed and revised the manuscript. TL contributed to the study design, reviewed and revised the manuscript. SAS conceptualised and designed the study together with SV, coordinated and supervised the database, reviewed and revised the manuscript.

Funding: This study was supported by the Unit of Research, Development, and Education, Östersund Hospital, Östersund, Sweden JLL-930202 (to SV); ALF Umeå University, Umeå, Sweden RV-933162 (to SAS).

Competing interests: We have no conflict of interest to declare.

Patient consent: Patient informed consent was not needed because the data were collected retrospectively and anonymised prior to analysis.

Ethics approval: The study was approved by the Regional Ethics Board in Umeå, reference number 2012-265-31M and 2017-399-32M.

Acknowledgement: The authors would like to thank Per E. Gustafsson, PhD at the Department of Epidemiology and Global Health, Umeå University, for reviewing and revising the manuscript and Johan Boström, MD at Department of Clinical Sciences, Pediatrics, Umeå University for help in creating the dataset. Håkan Sjöberg, MEc at Statistics Sweden, for help in compiling the dataset.

Data sharing statement: We used deidentified register data obtained from third parties. It includes sensitive information and some access restrictions may apply. Interested researchers need to obtain data directly from the National Board of Health and Welfare in Sweden (socialstyrelsen@socialstyrelsen.se) and Statistics Sweden (scb@scb.se).

Children included in the study were identified in the Medical Birth Register, data on hospitalisations were obtained from the Swedish National Patient Register and data on deaths were obtained from the Cause of Death Register. All of these registers are maintained by the National Board of Health

For peer review only

and Welfare in Sweden. Data on maternal education were obtained from the Longitudinal Integration Database for Health Insurance and Labour market Studies and data on migration were obtained from The Total Population Register, both registers are maintained by Statistics Sweden.

For peer review only

REFERENCES

1 Fleischmann-Struzek C, Goldfarb DM, Schlattmann P, Schlapbach LJ, Reinhart K, Kissoon N. The global burden of paediatric and neonatal sepsis: a systematic review. *Lancet Respir Med* 2018;6:223-30.
2 Collaborators GBDM. Global, regional, and national burden of meningitis, 1990-2016: a systematic analysis for the Global Burden of Disease Study 2016. *Lancet Neurol* 2018;17:1061-82.
3 Agyeman PKA, Schlapbach LJ, Giannoni E*, et al.* Epidemiology of blood culture-proven bacterial sepsis in children in Switzerland: a population-based cohort study. *Lancet Child Adolesc Health* 2017;1:124-33.
4 Schlapbach LJ, Straney L, Alexander J*, et al.* Mortality related to invasive infections, sepsis, and septic shock in critically ill children in Australia and New Zealand, 2002-13: a multicentre retrospective cohort study. *Lancet Infect Dis* 2015;15:46-54.
5 Ladhani SN, Collins S, Djennad A*, et al.* Rapid increase in non-vaccine serotypes causing invasive pneumococcal disease in England and Wales, 2000-17: a prospective national observational cohort study. *Lancet Infect Dis* 2018;18:441-51.
6 Weiss SL, Peters MJ, Alhazzani W*, et al.* Surviving sepsis campaign international guidelines for the management of septic shock and sepsis-associated organ dysfunction in children. *Intensive care medicine* 2020;46:10-67.
7 van de Beek D, Cabellos C, Dzupova O*, et al.* ESCMID guideline: diagnosis and treatment of acute bacterial meningitis. *Clinical microbiology and infection : the official publication of the European Society of Clinical Microbiology and Infectious Diseases* 2016;22 Suppl 3:S37-62.
8 National Institute for Health and Care Excellence. Fever in under 5s: assessment and initial management. 2019.
9 Puopolo KM, Benitz WE, Zaoutis TE, Committee On F, Newborn, Committee On Infectious D. Management of Neonates Born at >/=35 0/7 Weeks' Gestation With Suspected or Proven Early-Onset Bacterial Sepsis. *Pediatrics* 2018;142.
10 National Institute for Health and Care Excellence. Neonatal infection (early onset): antibiotics for prevention and treatment. 2012.
11 National Institute for Health and Care Excellence. Sepsis: recognition, diagnosis and early management. 2016.
12 Miller JE, Hammond GC, Strunk T*, et al.* Association of gestational age and growth measures at birth with infection-related admissions to hospital throughout childhood: a population-based, data-linkage study from Western Australia. *Lancet Infect Dis* 2016;16:952-61.
13 Hviid A, Melbye M. The impact of birth weight on infectious disease hospitalization in childhood. *American journal of epidemiology* 2007;165:756-61.
14 The Public Health Agency of Sweden. Tecken på allvarlig infektion hos barn. 2014.
15 The National Board of Health and Welfare in Sweden. The Swedish Medical Birth Register - A summary of content and quality. 2003.
16 Ludvigsson JF, Andersson E, Ekbom A*, et al.* External review and validation of the Swedish national inpatient register. *BMC public health* 2011;11:450.
17 Ludvigsson JF, Svedberg P, Olen O, Bruze G, Neovius M. The longitudinal integrated database for health insurance and labour market studies (LISA) and its use in medical research. *European journal of epidemiology* 2019;34:423-37.
18 Brooke HL, Talback M, Hornblad J*, et al.* The Swedish cause of death register. *European journal of epidemiology* 2017;32:765-73.
19 Ludvigsson JF, Lu D, Hammarstrom L, Cnattingius S, Fang F. Small for gestational age and risk of childhood mortality: A Swedish population study. *PLoS medicine* 2018;15:e1002717.
20 The National Board of Health and Welfare in Sweden. Registration of Congenital Malformations in the Swedish Health Registers. 2004.

For peer review only

21 Mattsson K, Kallen K, Rignell-Hydbom A*, et al.* Cotinine Validation of Self-Reported Smoking During Pregnancy in the Swedish Medical Birth Register. *Nicotine & tobacco research : official journal of the Society for Research on Nicotine and Tobacco* 2016;18:79-83.
22 Hanley JA, Negassa A, Edwardes MD, Forrester JE. Statistical analysis of correlated data using generalized estimating equations: an orientation. *American journal of epidemiology* 2003;157:364-75.
23 Hosmer DW, Lemeshow S. Confidence interval estimation of interaction. *Epidemiology* 1992;3:452-6.
24 VanderWeele TJ, Knol MJ. A Tutorial on Interaction. *Epidemiologic Methods* 2014;3:33-72.
25 Sterne JA, White IR, Carlin JB*, et al.* Multiple imputation for missing data in epidemiological and clinical research: potential and pitfalls. *Bmj* 2009;338:b2393.
26 Johansson Kostenniemi U, Norman D, Sellin M, Silfverdal SA. Sustained reductions of invasive infectious disease following general infant Haemophilus influenzae type b and pneumococcal vaccination in a Swedish Arctic region. *Acta paediatrica* 2019;108:1871-8.
27 Berglund A, Ekelund M, Fletcher MA, Nyman L. All-cause pneumonia hospitalizations in children <2 years old in sweden, 1998 to 2012: impact of pneumococcal conjugate vaccine introduction. *PloS one* 2014;9:e112211.
28 Kristensen K, Hjuler T, Ravn H, Simoes EA, Stensballe LG. Chronic diseases, chromosomal abnormalities, and congenital malformations as risk factors for respiratory syncytial virus hospitalization: a population-based cohort study. *Clinical infectious diseases : an official publication of the Infectious Diseases Society of America* 2012;54:810-7.
29 Muenchhoff M, Goulder PJ. Sex differences in pediatric infectious diseases. *The Journal of infectious diseases* 2014;209 Suppl 3:S120-6.
30 Yusuf HR, Rochat RW, Baughman WS*, et al.* Maternal cigarette smoking and invasive meningococcal disease: a cohort study among young children in metropolitan Atlanta, 1989-1996. *American journal of public health* 1999;89:712-7.
31 Aberg A, Westbom L. Association between maternal pre-existing or gestational diabetes and health problems in children. *Acta paediatrica* 2001;90:746-50.
32 Goldacre MJ, Wotton CJ, Maisonneuve JJ. Maternal and perinatal factors associated with subsequent meningococcal, Haemophilus or enteroviral meningitis in children: database study. *Epidemiology and infection* 2014;142:371-8.
33 Biering-Sorensen S, Sondergaard G, Vitting Andersen K, Andersen AM, Mortensen LH. Time trends in socio-economic factors and risk of hospitalisation with infectious diseases in pre-school children 1985-2004: a Danish register-based study. *Paediatric and perinatal epidemiology* 2012;26:226-35.
34 Baker MG, Barnard LT, Kvalsvig A*, et al.* Increasing incidence of serious infectious diseases and inequalities in New Zealand: a national epidemiological study. *Lancet* 2012;379:1112-9.
35 Wettergren B, Blennow M, Hjern A, Soder O, Ludvigsson JF. Child Health Systems in Sweden. *The Journal of pediatrics* 2016;177S:S187-S202.

For peer review only

Table 1: Birth, pregnancy and sociodemographic characteristics of the study population

	Included			Excluded*
	Sepsis	Bacterial meningitis	All included	
	n = 1011	n = 382	n = 1 406 547	n = 280 202
Sex:				
Male	55.8 (564)	57.9 (221)	51.4 (722 988)	51.4 (144 079)
Female	44.2 (447)	42.4 (162)	48.6 (683 559)	48.6 (136 117)
Gestation age†:				
Extremely preterm	2.5 (25)	1.0 (4)	0.1 (2012)	0.7 (1927)
Very preterm	2.4 (24)	2.1 (8)	0.4 (5457)	1.6 (4418)
Moderate preterm	8.8 (89)	5.8 (22)	4.0 (56 291)	10.3 (28 979)
Term	80.7 (816)	84.8 (324)	88.3 (1 242 234)	81.4 (227 950)
Post-term	5.6 (57)	6.5 (25)	7.1 (100 553)	5.7 (15 926)
SGA:				
Yes	7.8 (79)	3.7 (14)	2.1 (30 099)	2.1 (5972)
No	92.2 (932)	96.6 (369)	97.9 (1 376 448)	78.7 (220 473)
LGA:				
Yes	5.1 (52)	5.0 (19)	3.7 (51 816)	3.0 (8334)
No	94.9 (959)	95.3 (364)	96.3 (1 354 731)	77.8 (218 111)
Congenital malformation‡:				
Yes	14.1 (143)	6.0 (23)	3.4 (48 209)	3.7 (10 401)
No	85.9 (868)	94.2 (360)	96.6 (1 358 338)	96.3 (269 801)
Maternal age:				
Mean (SD)	30.0 (5.1)	29.7 (5.5)	30.1 (5.1)	30.0 (5.5)
Maternal smoking:				
No smoking	90.6 (916)	86.9 (332)	91.5 (1 286 708)	61.3 (171 790)
Smoking	9.4 (95)	13.4 (51)	8.5 (119 839)	6.9 (19 472)
Pregnancy BMI§:				
Underweight	2.1 (21)	2.4(9)	2.4 (33 836)	1.0 (2895)
Normal	59.8 (605)	60.7 (232)	61.6 (865 874)	20.3 (56 838)
Overweight	25.7 (260)	25.1 (96)	24.8 (348 695)	9.1 (25 600)
Obese	12.4 (125)	12.0 (46)	11.2 (158 142)	4.3 (12 030)
Parity:				
Mean (SD)	1.9 (1.0)	1.8 (1.1)	1.8 (1.0)	2.0 (1.2)
Maternal education in years:				
≤9	14.8 (150)	15.4 (59)	11.6 (162 814)	10.6 (29 682)
10–12	42.0 (425)	42.4 (162)	43.6 (613 370)	36.6 (102 593)
13–14	12.7 (127)	16.0 (61)	13.7 (192 659)	12.6 (35 362)
≥15	30.6 (309)	26.4 (101)	31.1 (437 704)	25.7 (71 907)
Maternal country of birth:				
Sweden	77.3 (782)	80.6 (308)	81.2 (1 142 682)	70.9 (198 735)
Other Nordic	1.8 (18)	1.8 (7)	1.7 (23 403)	2.6 (7173)
Other Europe and North America	8.8 (89)	6.8 (26)	6.2 (87 864)	9.0 (25 278)
Asia	8.6 (87)	6.3 (24)	7.4 (103 858)	10.8 (30 392)

For peer review only

Africa	2.4 (24)	3.4 (13)	2.4 (33 230)	5.2 (14 644)
Other	1.1 (11)	1.3 (5)	1.1 (15 510)	1.3 (3688)
Year of birth:				
1997–2002	27.1 (274)	33.0 (126)	29.5 (41 4783)	40.3 (112 908)
2003–2008	40.6 (410)	46.1 (176)	35.8 (503 841)	37.1 (103 988)
2009–2013	32.3 (327)	21.2 (81)	34.7 (487 923)	22.6 (63 306)

Values are percentages (numbers) unless stated otherwise.

*Children with missing data were excluded (17% of the original cohort). Data on variables were available in 35%–100% of the excluded children.

†Gestational age categorised as extremely premature (22–27 weeks), very premature (28–31 weeks), moderate premature (32–36 weeks), term (37–41 weeks) and post-term (≥42 weeks)

‡ICD-10 codes: Q00–Q99.

§BMI categorised as underweight (BMI<18.5 kg/m^2). normal (BMI 18.5–24.9 kg/m^2). overweight (BMI 25.0–29.9 kg/ m^2) and obese (BMI≥30.0 kg/m^2).

SGA, small for gestational age. LGA, large for gestational age. BMI, body mass index. SD, standard deviation. ICD-10, International Classification of Diseases, 10th revision

For peer review only

Table 2: Crude and adjusted analyses of the association between perinatal factors and hospitalisations for sepsis and bacterial meningitis in early childhood (28 days until 2 years of age)

	Sepsis		Bacterial meningitis	
	Crude IRR (95% CI)	Adjusted IRR (95% CI)	Crude IRR (95% CI)	Adjusted IRR (95% CI)
Sex:				
Male	1.12 (0.97–1.30)	1.09 (0.94–1.26)	1.35 (1.09–1.66)	1.33 (1.08–1.64)
Female	1 ref	1 ref	1 ref	1 ref
Gestational age*:				
Extremely preterm	15.73 (10.49–23.59)	10.37 (6.78–15.86)	7.50 (2.80–20.10)	6.22 (2.28–16.94)
Very preterm	6.44 (4.07–10.18)	4.06 (2.49–6.63)	5.99 (2.89–12.45)	4.95 (2.39–10.24)
Moderate preterm	2.17 (1.71–2.76)	1.85 (1.45–2.37)	1.41 (0.92–2.18)	1.31 (0.84–2.03)
Term	1 ref	1 ref	1 ref	1 ref
Post-term	0.80 (0.59–1.07)	0.80 (0.59–1.08)	0.93 (0.62–1.41)	0.90 (0.59–1.36)
SGA:				
Yes	3.51 (2.72–4.53)	2.44 (1.83–3.25)	1.89 (1.08–3.30)	1.36 (0.78–2.40)
No	1 ref	1 ref	1 ref	1 ref
LGA:				
Yes	1.69 (1.18–2.42)	1.71 (1.19–2.44)	1.43 (0.89–2.31)	1.51 (0.93–2.45)
No	1 ref	1 ref	1 ref	1 ref
Congenital malformation†:				
Yes	4.41 (3.61–5.40)	3.89 (3.17–4.77)	1.86 (1.21–2.88)	1.69 (1.09–2.62)
No	1 ref	1 ref	1 ref	1 ref
Maternal age:				
Per year of age	0.99 (0.98–1.01)	0.99 (0.97–1.00)	0.99 (0.96–1.01)	0.99 (0.97–1.02)
Maternal smoking:				
Yes	1.08 (0.85–1.38)	0.96 (0.75–1.25)	1.65 (1.22–2.24)	1.46 (1.05–2.04)
No	1 ref	1 ref	1 ref	1 ref
Pregnancy BMI‡:				
Underweight	0.84 (0.52–1.37)	0.77 (0.48–1.26)	0.95 (0.49–1.85)	0.88 (0.45–1.71)

For peer review only

Normal	1 ref	1 ref	1 ref	1 ref
Overweight	1.13 (0.95–1.33)	1.10 (0.92–1.30)	1.08 (0.84–1.39)	1.06 (0.82–1.36)
Obese	1.21 (0.96–1.52)	1.11 (0.88–1.39)	1.06 (0.77–1.46)	0.99 (0.72–1.37)
Parity:				
Per child	1.05 (0.98–1.13)	1.08 (1.00–1.16)	0.98 (0.87–1.10)	0.96 (0.85–1.08)
Maternal education in years:				
≤9	1.27 (1.01–1.59)	1.07 (0.83–1.39)	1.70 (1.23–2.36)	1.39 (0.95–2.02)
10–12	0.95 (0.80–1.13)	0.89 (0.74–1.07)	1.23 (0.96–1.59)	1.09 (0.83–1.43)
13–14	0.93 (0.73–1.18)	0.90 (0.71–1.15)	1.46 (1.06–2.02)	1.37 (0.98–1.90)
≥15	1 ref	1 ref	1 ref	1 ref
Maternal country of birth:				
Sweden	1 ref	1 ref	1 ref	1 ref
Other Nordic	1.03 (0.62–1.72)	1.01 (0.61–1.69)	1.23 (0.56–2.70)	1.18 (0.54–2.58)
Other Europe and North America	1.36 (1.07–1.72)	1.33 (1.05–1.70)	1.09 (0.73–1.64)	1.10 (0.73–1.66)
Asia	1.24 (0.96–1.61)	1.17 (0.90–1.53)	0.92 (0.60–1.42)	0.94 (0.61–1.46)
Africa	1.12 (0.65–1.94)	1.00 (0.57–1.75)	1.50 (0.84–2.65)	1.60 (0.91–2.80)
Other	0.88 (0.47–1.62)	0.85 (0.46–1.57)	1.37 (0.54–3.49)	1.38 (0.54–3.52)
Year of birth:				
1997–2002	1 ref	1 ref	1 ref	1 ref
2003–2008	1.26 (1.05–1.50)	1.23 (1.03–1.48)	1.18 (0.93–1.49)	1.23 (0.96–1.57)
2009–2013	1.00 (0.83–1.20)	0.98 (0.80–1.19)	0.54 (0.41–0.72)	0.57 (0.42–0.77)
Observations (N):	1 406 547	1 406 547	1 406 547	1 406 547
Clusters (Maternal ID):	891 950	891 950	891 950	891 950

Analyses excluded children with missing data (n = 280 202), leaving 1 406 547 children. Adjusted analyses were controlled for sex, gestational age, SGA, LGA, congenital malformation, maternal age, smoking during pregnancy, pregnancy BMI, parity, maternal education level, maternal country of birth and year of birth.

For peer review only

*Gestational age categorised as extremely premature (22–27 weeks), very premature (28–31 weeks), moderate premature (32–36 weeks), term (37–41 weeks) and post-term (≥42 weeks)

†ICD-10 codes: Q00–Q99.

‡BMI categorised as underweight (BMI<18.5 kg/m^2), normal (BMI 18.5–24.9 kg/m^2), overweight (BMI 25.0–29.9 kg/m^2) and obese (BMI≥30.0 kg/m^2).

IRR, incidence rate ratio. CI, confidence interval. SGA, small for gestational age. LGA, large for gestational age. BMI, body mass index. ICD-10, International Classification of Diseases, 10th revision.

For peer review only

Table 3: Interactions between sex, prematurity and perinatal factors

	Sepsis	Bacterial meningitis
RERI with sex*:	RERI (95% CI)	RERI (95% CI)
Prematurity*sex	-0.78 (-1.78–0.22)	1.14 (-0.24–2.53)
SGA*sex	-0.45 (-1.87–0.95)	1.83 (-0.08–3.73)
LGA*sex	0.04 (-1.20–1.27)	1.11 (-0.49–2.71)
Congenital malformations*sex	-0.46 (-2.06–1.13)	-0.96 (-2.80–0.88)
Maternal smoking*sex	-0.56 (-1.37–0.25)	0.07 (-0.61–0.75)
RERI with prematurity†:		
SGA*prematurity	1.00 (-1.36–3.36)	0.36 (-2.73–3.45)
LGA*prematurity	-0.97 (-3.01–1.08)	1.58 (-2.76–5.91)
Congenital malformations*prematurity	7.75 (3.45–12.06)	-0.33 (-3.19–2.53)
Maternal smoking*prematurity	-0.51 (-2.12–1.11)	-0.09 (-2.33–2.16)

Analyses excluded children with missing data (n = 280 202), leaving 1 406 547 children. Analyses were controlled for sex, gestational age, SGA, LGA, congenital malformation, maternal age, smoking during pregnancy, pregnancy BMI, parity, maternal education level, maternal country of birth and year of birth. Gestational age was included as a binary variable (0 = gestational age ≥37 weeks, 1 = gestational age <37 weeks); other variables were included as previously described.

*Female is the reference category.

†Gestational age ≥37 weeks is the reference category.

RERI, relative excess risk due to interaction. CI, confidence interval. SGA, small for gestational age. LGA, large for gestational age.

For peer review only

Figure 1. Flowchart of the study population. Complete case analyses included all children in the study population. Multiple imputation analyses included all children in the target population. *Children with missing data on any variable (sex, gestational age, small for gestational age, large for gestational age, congenital malformation, maternal age, smoking during pregnancy, pregnancy body mass index, parity, maternal education level, maternal country of birth and year of birth) were excluded.

Figure 2. Incidence of sepsis (brown circles) and bacterial meningitis (orange squares) in early childhood, by age in months. Incidence rates calculated as the number of hospital admissions per 100 000 person-years at risk. Vertical lines represent 95% confidence intervals. Analyses included 1 406 547 children.

Figure 3. Incidence of sepsis (brown circles) and bacterial meningitis (orange squares) in early childhood, by year of birth. Incidence rates calculated as the number of hospital admissions per 100 000 person-years at risk. Vertical lines represent 95% confidence intervals. Analyses included 1 406 547 children.

For peer review only

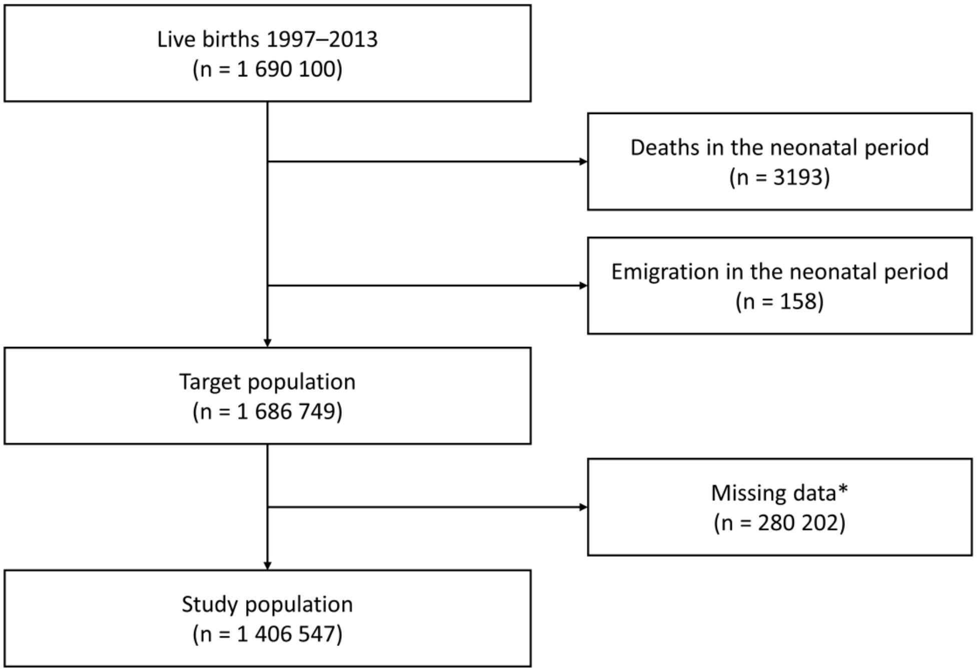

Figure 1. Flowchart of the study population. Complete case analyses included all children in the study population. Multiple imputation analyses included all children in the target population. *Children with missing data on any variable (sex, gestational age, small for gestational age, large for gestational age, congenital malformation, maternal age, smoking during pregnancy, pregnancy body mass index, parity, maternal education level, maternal country of birth and year of birth) were excluded.

2021x1417mm (38 x 38 DPI)

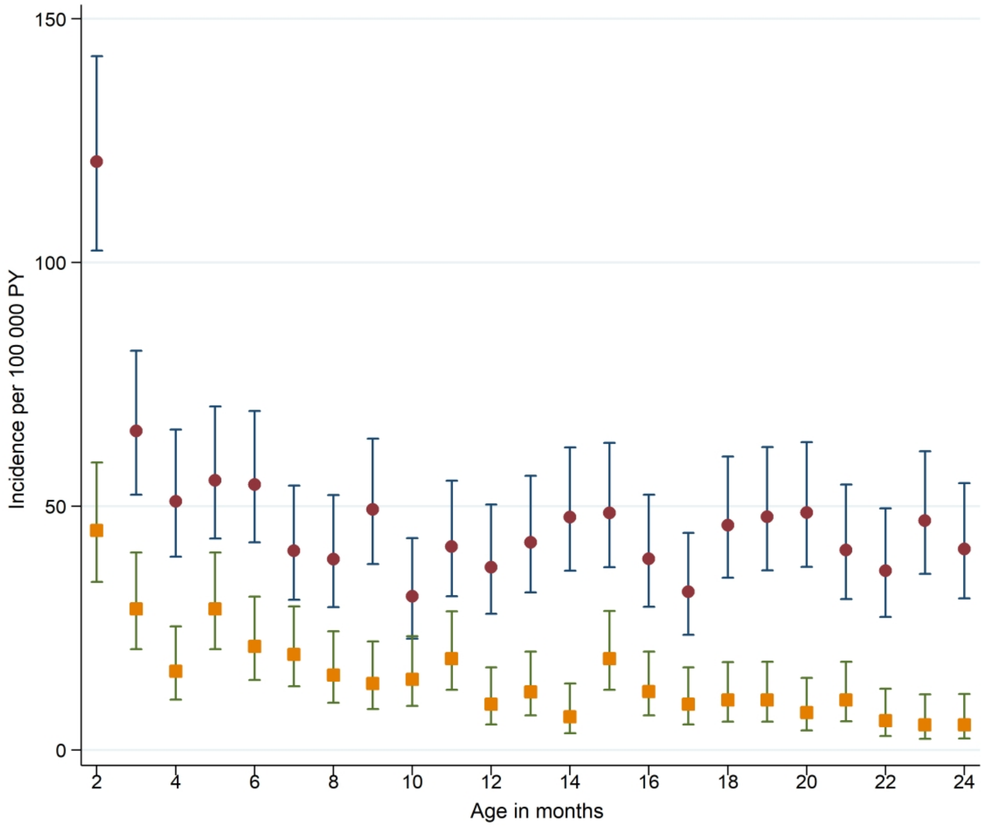

Figure 2. Incidence of sepsis (brown circles) and bacterial meningitis (orange squares) in early childhood, by age in months. Incidence rates calculated as the number of hospital admissions per 100 000 person-years at risk. Vertical lines represent 95% confidence intervals. Analyses included 1 406 547 children.

1909x1593mm (38 x 38 DPI)

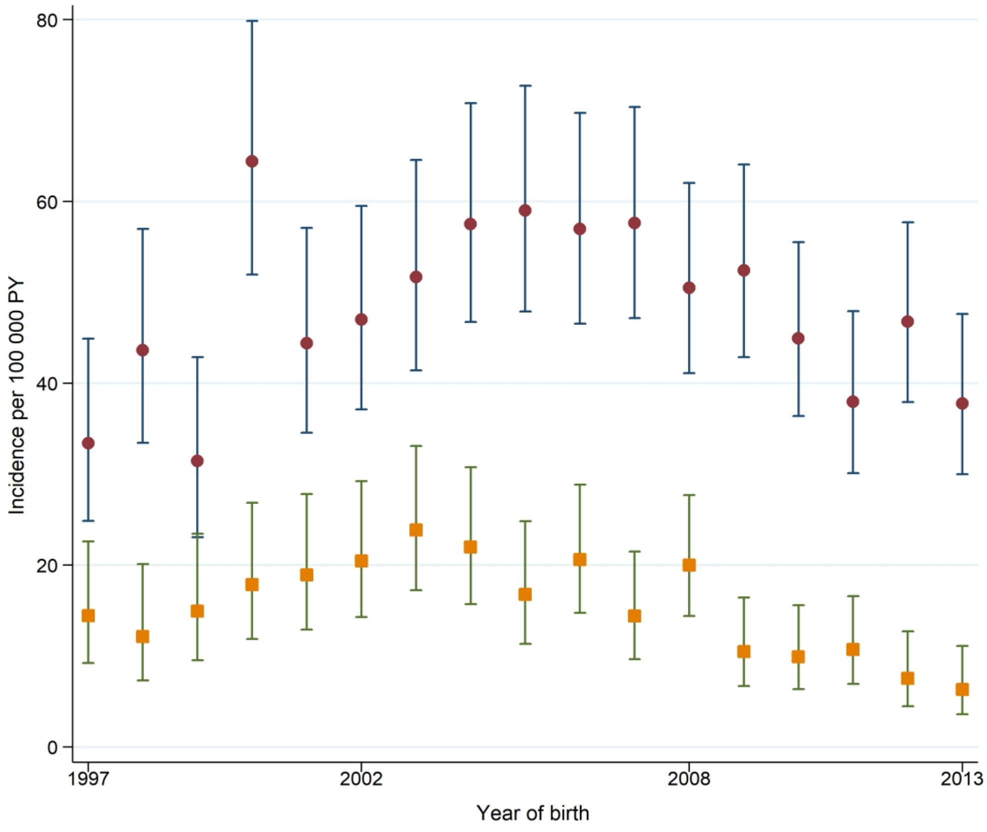

Figure 3. Incidence of sepsis (brown circles) and bacterial meningitis (orange squares) in early childhood, by year of birth. Incidence rates calculated as the number of hospital admissions per 100 000 person-years at risk. Vertical lines represent 95% confidence intervals. Analyses included 1 406 547 children.

1919x1620mm (38 x 38 DPI)

Appendix A

Table A: Incidence overall and for categories of bacterial meningitis in early childhood (28 days until 2 years of age), by year of birth

	1997–2002	2003–2008	2009–2013
	Incidence (95% CI)	Incidence (95% CI)	Incidence (95% CI)
All	16.6 (14.0–19.7)	19.6 (17.0–22.6)	9.0 (7.2–11.1)
Meningococcal meningitis*	2.5 (1.6–3.9)	1.6 (0.9–2.6)	1.2 (0.7–2.1)
Haemophilus meningitis†	1.0 (0.5–2.0)	1.0 (0.6–1.9)	0.7 (0.4–1.6)
Pneumococcal meningitis‡	8.3 (6.5–10.6)	10.0 (8.2–12.2)	2.2 (1.5–3.4)
Streptococcus meningitis§	1.5 (0.9–2.7)	1.7 (1.0–2.7)	1.5 (0.9–2.5)
Other meningitis¶	3.3 (2.2–4.8)	5.3 (4.0–6.9)	3.3 (2.3–4.7)

Incidence rates estimated as the number of hospital admissions per 100 000 person-years at risk.
Analyses included 1 406 547 children.
*ICD-10 code A39.0
†ICD-10 code G00.0
‡ICD-10 code G00.1
§ICD-10 code G00.2
¶ICD-10 codes G00.3-G00.8 and A32.1
CI, confidence intervals. ICD-10, International Classification of Diseases, 10th revision.

For peer review only

Appendix B

Table A: Crude and adjusted analyses of the association between perinatal factors and hospitalisations for sepsis and bacterial meningitis in early childhood (28 days until 2 years of age), estimated using multiple imputation

	Sepsis		Bacterial meningitis	
	Crude IRR (95% CI)	Adjusted IRR (95% CI)	Crude IRR (95% CI)	Adjusted IRR (95% CI)
Sex:				
Male	1.07 (0.94–1.22)	1.04 (0.91–1.18)	1.38 (1.14–1.66)	1.36 (1.13–1.64)
Female	1 ref	1 ref	1 ref	1 ref
Gestational age*:				
Extremely preterm	14.83 (10.84–20.30)	9.91 (7.09–13.84)	13.19 (7.23–24.07)	10.85 (5.77–20.41)
Very preterm	5.90 (4.24–8.22)	3.93 (2.74–5.63)	5.06 (2.87–8.94)	4.03 (2.27–7.16)
Moderate preterm	2.50 (2.01–3.10)	2.16 (1.73–2.69)	2.00 (1.45–2.76)	1.84 (1.33–2.55)
Term	1 ref	1 ref	1 ref	1 ref
Post-term	0.80 (0.61–1.04)	0.80 (0.61–1.05)	0.92 (0.62–1.34)	0.88 (0.60–1.30)
SGA:				
Yes	3.47 (2.75–4.37)	2.18 (1.69–2.83)	2.53 (1.61–3.97)	1.63 (1.02–2.62)
No	1 ref	1 ref	1 ref	1 ref
LGA:				
Yes	1.61 (1.17–2.20)	1.61 (1.17–2.21)	1.29 (0.83–2.01)	1.36 (0.87–2.13)
No	1 ref	1 ref	1 ref	1 ref
Congenital malformation†:				
Yes	4.68 (3.95–5.55)	3.96 (3.32–4.71)	2.01 (1.40–2.89)	1.70 (1.18–2.45)
No	1 ref	1 ref	1 ref	1 ref
Maternal age:				
Per year of age	0.99 (0.98–1.00)	0.98 (0.97–1.00)	0.98 (0.97–1.00)	0.99 (0.97–1.01)
Maternal smoking:				
Yes	1.16 (0.94–1.45)	1.00 (0.79–1.26)	1.68 (1.27–2.23)	1.47 (1.08–2.01)
No	1 ref	1 ref	1 ref	1 ref

For peer review only

Pregnancy BMI‡:				
Underweight	0.81 (0.51–1.29)	0.73 (0.46–1.16)	0.90 (0.46–1.74)	0.81 (0.42–1.57)
Normal	1 ref	1 ref	1 ref	1 ref
Overweight	1.12 (0.96–1.32)	1.09 (0.93–1.28)	1.09 (0.85–1.39)	1.05 (0.83–1.35)
Obese	1.24 (1.00–1.53)	1.11 (0.90–1.38)	1.07 (0.79–1.45)	0.98 (0.72–1.34)
Parity:				
Per child	1.06 (1.01–1.12)	1.09 (1.02–1.15)	1.01 (0.92–1.11)	1.00 (0.90–1.10)
Maternal education in years:				
≤9	1.41 (1.15–1.72)	1.15 (0.90–1.46)	1.72 (1.29–2.29)	1.33 (0.95–1.87)
10–12	1.00 (0.85–1.17)	0.91 (0.77–1.09)	1.23 (0.98–1.55)	1.07 (0.83–1.37)
13–14	0.96 (0.77–1.18)	0.93 (0.75–1.16)	1.42 (1.06–1.89)	1.33 (0.99–1.78)
≥15	1 ref	1 ref	1 ref	1 ref
Maternal country of birth:				
Sweden	1 ref	1 ref	1 ref	1 ref
Other Nordic	1.22 (0.79–1.91)	1.20 (0.76–1.87)	1.13 (0.57–2.25)	1.08 (0.54–2.14)
Other Europe and North America	1.43 (1.16–1.76)	1.39 (1.13–1.70)	1.10 (0.78–1.56)	1.08 (0.76–1.53)
Asia	1.20 (0.96–1.49)	1.10 (0.88–1.38)	0.97 (0.68–1.40)	0.97 (0.67–1.39)
Africa	1.20 (0.82–1.75)	1.03 (0.69–1.53)	1.54 (0.98–2.41)	1.57 (1.00–2.46)
Other	0.99 (0.56–1.75)	0.96 (0.54–1.70)	1.93 (0.96–3.87)	1.91 (0.95–3.86)
Year of birth:				
1997–2002	1 ref	1 ref	1 ref	1 ref
2003–2008	1.20 (1.02–1.40)	1.20 (1.02–1.41)	1.23 (1.00–1.52)	1.29 (1.04–1.59)
2009–2013	1.00 (0.85–1.18)	1.00 (0.84–1.19)	0.58 (0.45–0.75)	0.61 (0.47–0.80)
Observations (N):	1 406 547	1 406 547	1 406 547	1 406 547
Clusters (Maternal ID):	891 950	891 950	891 950	891 950

Multiple imputation models included all 1 686 749 children. Adjusted analyses were controlled for sex, gestational age, small for gestational age, large for gestational age, congenital malformation, maternal age, smoking during pregnancy, pregnancy BMI, parity, maternal education level, maternal country of birth and year of birth.

For peer review only

*Gestational age categorised as extremely premature (22–27 weeks), very premature (28–31 weeks), moderate premature (32–36 weeks), term (37–41 weeks) and post-term (≥42 weeks)
†ICD-10 codes: Q00–Q99.
‡BMI categorised as underweight (BMI<18.5 kg/m^2), normal (BMI 18.5–24.9 kg/m^2), overweight (BMI 25.0–29.9 kg/m^2) and obese (BMI≥30.0 kg/m^2).
IRR, incidence rate ratio. CI, confidence intervals. SGA, small for gestational age. LGA, large for gestational age. BMI, body mass index. ICD-10, International Classification of Diseases, 10th revision.

For peer review only

STROBE Statement—Checklist of items that should be included in reports of ***cohort studies***

	Item No	Recommendation	Page No
**Title and abstract**	1	(*a*) Indicate the study's design with a commonly used term in the title or the abstract	1
		(*b*) Provide in the abstract an informative and balanced summary of what was done and what was found	2
**Introduction**			
Background/rationale	2	Explain the scientific background and rationale for the investigation being reported	4
Objectives	3	State specific objectives, including any prespecified hypotheses	4
**Methods**			
Study design	4	Present key elements of study design early in the paper	5
Setting	5	Describe the setting, locations, and relevant dates, including periods of recruitment, exposure, follow-up, and data collection	5
Participants	6	(*a*) Give the eligibility criteria, and the sources and methods of selection of participants. Describe methods of follow-up	5 + fig 1
		(*b*) For matched studies, give matching criteria and number of exposed and unexposed	
Variables	7	Clearly define all outcomes, exposures, predictors, potential confounders, and effect modifiers. Give diagnostic criteria, if applicable	5-6
Data sources/ measurement	8*	For each variable of interest, give sources of data and details of methods of assessment (measurement). Describe comparability of assessment methods if there is more than one group	5-6
Bias	9	Describe any efforts to address potential sources of bias	7
Study size	10	Explain how the study size was arrived at	-
Quantitative variables	11	Explain how quantitative variables were handled in the analyses. If applicable, describe which groupings were chosen and why	5-6
Statistical methods	12	(*a*) Describe all statistical methods, including those used to control for confounding	6-7
		(*b*) Describe any methods used to examine subgroups and interactions	
		(*c*) Explain how missing data were addressed	
		(*d*) If applicable, explain how loss to follow-up was addressed	
		(*e*) Describe any sensitivity analyses	
**Results**			
Participants	13*	(a) Report numbers of individuals at each stage of study—eg numbers potentially eligible, examined for eligibility, confirmed eligible, included in the study, completing follow-up, and analysed	7-8
		(b) Give reasons for non-participation at each stage	
		(c) Consider use of a flow diagram	
Descriptive data	14*	(a) Give characteristics of study participants (eg demographic, clinical, social) and information on exposures and potential confounders	8 + Table 1
		(b) Indicate number of participants with missing data for each variable of interest	Table 1
		(c) Summarise follow-up time (eg, average and total amount)	8
Outcome data	15*	Report numbers of outcome events or summary measures over time	8

For peer review only

For peer review only - http://bmjopen.bmj.com/site/about/guidelines.xhtml

Main results	16	(*a*) Give unadjusted estimates and, if applicable, confounder-adjusted estimates and their precision (eg, 95% confidence interval). Make clear which confounders were adjusted for and why they were included<br>(*b*) Report category boundaries when continuous variables were categorized<br>(*c*) If relevant, consider translating estimates of relative risk into absolute risk for a meaningful time period	8-9<br>Table2
Other analyses	17	Report other analyses done—eg analyses of subgroups and interactions, and sensitivity analyses	Table3 Appendix B
**Discussion**			
Key results	18	Summarise key results with reference to study objectives	9
Limitations	19	Discuss limitations of the study, taking into account sources of potential bias or imprecision. Discuss both direction and magnitude of any potential bias	11-12
Interpretation	20	Give a cautious overall interpretation of results considering objectives, limitations, multiplicity of analyses, results from similar studies, and other relevant evidence	9-11
Generalisability	21	Discuss the generalisability (external validity) of the study results	12
**Other information**			
Funding	22	Give the source of funding and the role of the funders for the present study and, if applicable, for the original study on which the present article is based	13

*Give information separately for exposed and unexposed groups.

Note: An Explanation and Elaboration article discusses each checklist item and gives methodological background and published examples of transparent reporting. The STROBE checklist is best used in conjunction with this article (freely available on the Web sites of PLoS Medicine at http://www.plosmedicine.org/, Annals of Internal Medicine at http://www.annals.org/, and Epidemiology at http://www.epidem.com/). Information on the STROBE Initiative is available at http://www.strobe-statement.org.

For peer review only

BMJ Open

Perinatal factors and hospitalisations for severe childhood infections: a population-based cohort study in Sweden

Journal:	*BMJ Open*
Manuscript ID	bmjopen-2021-054083.R2
Article Type:	Original research
Date Submitted by the Author:	20-Sep-2021
Complete List of Authors:	Videholm, Samuel; Umea University, Department of Clinical Sciences, Pediatrics<br>Kostenniemi, Urban; Umea Universitet, Department of Clinical Sciences, Pediatrics; Umea University, Department of Clinical Microbiology, Infectious Diseases<br>Lind, Torbjörn ; Umea University, Department of Clinical Sciences, Pediatrics<br>Silfverdal, Sven-Arne; Umea University, Department of Clinical Sciences, Pediatrics
**Primary Subject Heading**:	Epidemiology
Secondary Subject Heading:	Infectious diseases
Keywords:	INFECTIOUS DISEASES, Epidemiology < TROPICAL MEDICINE, Paediatric infectious disease & immunisation < PAEDIATRICS, Paediatric A&E and ambulatory care < PAEDIATRICS

SCHOLARONE™
Manuscripts

I, the Submitting Author has the right to grant and does grant on behalf of all authors of the Work (as defined in the below author licence), an exclusive licence and/or a non-exclusive licence for contributions from authors who are: i) UK Crown employees; ii) where BMJ has agreed a CC-BY licence shall apply, and/or iii) in accordance with the terms applicable for US Federal Government officers or employees acting as part of their official duties; on a worldwide, perpetual, irrevocable, royalty-free basis to BMJ Publishing Group Ltd ("BMJ") its licensees and where the relevant Journal is co-owned by BMJ to the co-owners of the Journal, to publish the Work in this journal and any other BMJ products and to exploit all rights, as set out in our licence.

The Submitting Author accepts and understands that any supply made under these terms is made by BMJ to the Submitting Author unless you are acting as an employee on behalf of your employer or a postgraduate student of an affiliated institution which is paying any applicable article publishing charge ("APC") for Open Access articles. Where the Submitting Author wishes to make the Work available on an Open Access basis (and intends to pay the relevant APC), the terms of reuse of such Open Access shall be governed by a Creative Commons licence – details of these licences and which Creative Commons licence will apply to this Work are set out in our licence referred to above.

Other than as permitted in any relevant BMJ Author's Self Archiving Policies, I confirm this Work has not been accepted for publication elsewhere, is not being considered for publication elsewhere and does not duplicate material already published. I confirm all authors consent to publication of this Work and authorise the granting of this licence.

For peer review only

Perinatal factors and hospitalisations for severe childhood infections: a population-based cohort study in Sweden

Samuel Videholm, MD[1], Urban Johansson Kostenniemi, MD, PhD[1,2], Torbjörn Lind, MD, PhD[1], Sven-Arne Silfverdal, MD, PhD, MPH[1]

[1]Department of Clinical Sciences, Pediatrics, Umeå University, Umeå, Sweden

[2]Department of Clinical Microbiology, Infectious Diseases, Umeå University, Umeå, Sweden

Corresponding Author:

Name: Samuel Videholm

E-mail: samuel.videholm@umu.se

Telephone number: +46 907858919

Fax: +46 90123728

Postal address:

Department of Clinical Sciences, Pediatrics,

Umeå University

901 87 Umeå

Sweden

Keywords:

Epidemiology

Infectious Diseases

Paediatric infectious disease & immunization

Paediatric A&E and ambulatory care

Word count: 2804

For peer review only

Abstract

Objective: To examine the association between perinatal factors and hospitalisations for sepsis and bacterial meningitis in early childhood (from 28 days until 2 years of age).

Design: A population-based cohort study. The Swedish Medical Birth Register was combined with the National Inpatient Register, the Cause of Death Register, the Total Population Register and the Longitudinal integration database for health insurance and labour market studies. Associations between perinatal factors and hospitalisations were examined using negative binomial regression models.

Setting: Sweden.

Participants: 1 406 547 children born in Sweden between 1997 and 2013.

Main outcome measures: Hospital admissions for sepsis and bacterial meningitis recorded between 28 days and 2 years of life.

Results: Gestational age was inversely associated with severe infections i.e. extreme prematurity was strongly associated with an increased risk of sepsis, adjusted incidence rate ratio (aIRR) 10.37 (95% CI 6.78 to 15.86) and meningitis aIRR 6.22 (95% CI 2.28 to 16.94). Presence of congenital malformation was associated with sepsis aIRR 3.89 (95% CI 3.17 to 4.77) and meningitis aIRR 1.69 (95% CI 1.09 to 2.62). Moreover, children born small or large for gestational age were more likely to be hospitalised for sepsis and children exposed to maternal smoking were more likely to be hospitalised for meningitis.

Conclusions: Prematurity and several other perinatal factors were associated with an increased risk of severe infections in young children. Therefore, clinical guidelines for risk assessment of infections in young children should consider perinatal factors.

For peer review only

Strengths and limitations of this study:

The large number of events allowed us to estimate incidence rates of sepsis and bacterial meningitis by age in months and year of birth.

The linkage of high-quality health and administrative registers enabled us to examine key birth, pregnancy and sociodemographic characteristics.

Hospitalisations were identified using de-identified hospital discharge data; infections could not be confirmed by laboratory findings.

Information on perinatal factors was missing for 17% of children; nevertheless, results from analyses with imputed data were overall consistent with results from analyses restricted to children with complete data.

For peer review only

BACKGROUND

Sepsis and bacterial meningitis are major causes of morbidity and mortality in young children. After the neonatal period (from birth until 28 days), the global incidence of paediatric sepsis was estimated to be 1.2 million cases per year in 2013 and the global incidence of pediatric meningitis was around 280 000 cases per year in 2016.[1 2] In high-income countries, sepsis and bacterial meningitis still contribute significantly to the burden of childhood disease.[3-5]

Sepsis and bacterial meningitis are critical illnesses requiring early identification and rapid management, i.e. antibiotic treatment.[6 7] Early identification is challenging; clinical guidelines recommend that clinicians use a combination of symptoms, vital signs and laboratory findings.[7 8] In the neonatal period, clinicians are also recommended to consider perinatal risk factors including gestational age, maternal intraamniotic infection and maternal group B streptococcus colonisation.[9-11] After the neonatal period, children with certain perinatal conditions, e.g. low gestational age and low birth weight are known to be more susceptible to severe infections.[12 13] However, perinatal risk factors are not regularly considered in clinical guidelines for severe infections after the neonatal period.[7 8 11 14]

Therefore, we examined associations between perinatal factors, i.e. birth, pregnancy and sociodemographic characteristics, and hospitalisations for sepsis and bacterial meningitis in early childhood (from 28 days until 2 years of age).

METHODS

Patient and Public Involvement

This study used retrospectively collected register data. Therefore, patients or members of the public were not involved in the design or conduct of this study.

Study population

For peer review only

We conducted a population-based cohort study including children born in Sweden between 1997 and 2013, who resided in Sweden at 28 days of age. National health and administrative registers were combined to a dataset using the national registration number, a unique personal identification number assigned to all Swedish residents. The dataset included information from the Medical Birth Register which covers over 98-99% of all births and contains information on prenatal, delivery and neonatal care, the National Inpatient Register which holds information on 99% of all inpatient hospital admissions, the Longitudinal integration database for health, insurance and labour market studies which contains socio-economic data, the Cause of Death Register which covers over 97% of all deaths and the Total Population Register.[15-18] The data were anonymised and linked by the Swedish National Board of Health and Welfare.

Main Exposures

Information on pregnancy and birth characteristics were obtained from the Medical Birth Register. Gestational age was estimated using foetal ultrasound measurements in the early second trimester (performed in around 95% of pregnancies) or information of the last menstrual period and categorised as extremely premature (22–27 weeks), very premature (28–31 weeks), moderate premature (32–36 weeks), term (37–41 weeks) and post-term (≥42 weeks).[19] Sex was categorised as male or female. Small for gestational age (SGA) was defined as a birth weight below the 10th percentile for gestational age at birth.[19] Large for gestational age (LGA) was defined as a birthweight over the 90th percentile for gestational age at birth. Congenital malformation included abnormalities detected during the first 28 days; these malformations were recorded using International Classification of Diseases, 10th revision (ICD-10) codes Q00–Q99.[20] Maternal age was defined as age at delivery. Maternal smoking during pregnancy was self-reported (yes or no) during the first antenatal care visit, normally between 8 and 12 weeks of gestation.[21] Maternal body mass index (BMI) during pregnancy was calculated from weight measured at the first antenatal care visit and self-reported height, and categorised as underweight (<18.5 kg/m^2), normal (18.5–24.9 kg/m^2),

For peer review only - http://bmjopen.bmj.com/site/about/guidelines.xhtml

overweight (25.0–29.9 kg/m^2) and obese (≥30.0 kg/m^2).[15] Parity was defined as the number of live births including the index child.

Information on maternal education level was retrieved from the Longitudinal integration database for health, insurance and labour market studies and categorised as secondary school or less (≤9 years), upper secondary school (10–12 years), short postsecondary education (13–14 years) and long post-secondary education (≥15 years).

Information on maternal country of birth was obtained from the Total Population Register and categorised as Sweden, Other Nordic, Other Europe and North America, Asia, Africa and Other. The Total Population Register was also used to identify children who migrated and the Cause of Death Register was used to identify children who died.

Outcomes

The two main outcomes were number of hospitalisations for sepsis and number of hospitalisations for bacterial meningitis. Information on hospital admissions with a principal diagnosis of sepsis (ICD-10 codes: A39.1-A39.9, A40, A41, A48.3, A02.1 and A32.7) and/or bacterial meningitis (ICD-10 codes: A39.0, A32.1 and G00) were retrieved from the National Inpatient Register. Only hospitalisations with admission dates between 28 days and 2 years of age were included. Consequently, infections with admission date in the neonatal period were not included. Readmissions within 30 days were excluded.

Statistical analysis

We estimated incidence rates of sepsis and bacterial meningitis as the number of hospital admissions per 100 000 person-years (PY) at risk.

We examined associations between birth (sex, gestational age, SGA, LGA and congenital malformation), pregnancy (smoking during pregnancy, pregnancy BMI, and parity) and sociodemographic (maternal age, maternal education level and maternal country of birth)

For peer review only

characteristics, and hospitalisation for sepsis and bacterial meningitis using negative binomial regression models. Crude and adjusted analyses were performed for all variables. In the adjusted analyses, all analyses were adjusted for birth characteristics, pregnancy characteristics, sociodemographic characteristics and time trends (year of birth: 1997–2002, 2003–2008 and 2009–2013). All models were restricted to observations with complete data on all variables. Log follow up time (in days) was used as offset. Clustering within families was accounted for using generalized estimating equations with robust standard errors.[22] Results were presented as adjusted incidence rate ratios (aIRRs) with 95% confidence intervals (CIs).

In interaction analysis, we examined interactions between sex and prematurity (gestational age <37 weeks), SGA, LGA, congenital malformation and maternal smoking; between prematurity and SGA, LGA, congenital malformation and maternal smoking. Interactions were estimated on an additive scale by calculating the relative excess risk due to interaction (RERI). RERI was calculated using the following formula: $RERI = aIRR_{11} – aIRR_{10} – aIRR_{01} + 1$; 95% CIs were estimated using the delta method. [23 24]

In sensitivity analysis, we imputed missing data using multiple imputation.[25] The predictive model included all variables in the substantive models and both outcomes (hospitalisation for sepsis or bacterial meningitis). Variables were imputed in order of increasing missingness using logistic regression models (categorical variables) and linear regression models (continuous variables). Ten imputed datasets were generated.

All statistical analyses were performed using Stata Statistical Software: Release 14. College Station, Texas, USA.

Ethics

The study was approved by the Regional Ethics Board in Umeå, reference number 2012-265-31M and 2017-399-32M.

For peer review only

RESULTS

Study Population

The Medical Birth Register included 1 690 100 live births between 1997 and 2013. Children who died (n = 3193) or emigrated (n = 158) in the neonatal period were removed, leaving 1 686 749 children who resided in Sweden at 28 days of age (the target population). We excluded children with missing data (n = 280 202), leaving 1 406 547 children (figure 1). All children were followed until 2 years of age or censoring due to death (n = 1417) or emigration (n = 7185). In total, the study included 2 698 039 PY of follow-up time, 1298 hospitalisations for sepsis and 405 hospitalisations for bacterial meningitis.

Table 1 presents background characteristics of the study population. During the study period, 1011 children were hospitalised at least once for sepsis, 382 children were hospitalised at least once for bacterial meningitis and 18 children were hospitalised at least once for both sepsis and bacterial meningitis. Children hospitalised for severe infections were more likely to have certain characteristics, e.g. premature birth, small for gestational age and low maternal education level.

Incidence rates

Figure 2 shows the incidence rates of sepsis and bacterial meningitis hospitalisations by age in months. The incidence rate of sepsis decreased until the 3rd month of life. Thereafter, it remained around 45 hospitalisations per 100 000 PY. The incidence rate of bacterial meningitis decreased until the 4th month of life, thereafter it was around 20 hospitalisations per 100 000 PY in the first year of life and around 10 hospitalisations per 100 000 PY in the second year of life.

Figure 3 shows the incidence rates of sepsis and bacterial meningitis hospitalisations by year of birth. The incidence rate of bacterial meningitis was around 20 hospitalisations per 100 000 PY in children born 1997–2008. Thereafter, the incidence rate of bacterial meningitis decreased markedly. This was mainly due to a lower incidence rate of pneumococcal meningitis. Online supplementary appendix A

For peer review only

includes information on incidence rates for categories of bacterial meningitis grouped by year of birth.

Perinatal factors

Table 2 presents associations between perinatal factors and hospitalisations for sepsis and bacterial meningitis. Gestational age was inversely associated with severe infections. In comparison with term children, children born extremely preterm were much more likely to be hospitalised for sepsis aIRR 10.37 (95% CI 6.78 to 15.86) and bacterial meningitis aIRR 6.22 (95% CI 2.28 to 16.94). Presence of congenital malformation was associated with both sepsis aIRR 3.89 (95% CI 3.17 to 4.77) and bacterial meningitis aIRR 1.69 (95% CI 1.09 to 2.62). An increased risk of sepsis was observed in children born small for gestational age aIRR 2.44 (95% CI 1.83 to 3.25) or large for gestational age aIRR 1.71 (95% CI 1.19 to 2.44). Children born to mothers who smoked during pregnancy were more likely to be hospitalised for bacterial meningitis aIRR 1.46 (95% CI 1.05 to 2.04). Low maternal education (≤9 years) was in comparison with post-secondary education (≥15 years) associated with both sepsis and bacterial meningitis in the unadjusted analyses. Sensitivity analysis showed similar estimates after multiple imputation, except for a stronger association between extreme prematurity and meningitis. Online supplementary appendix B includes results from multiple imputation models.

Table 3 presents results from interaction analyses. An additive interaction was found between prematurity (gestational age <37 weeks) and congenital malformations on the risk of sepsis RERI 7.75 (95% CI 3.45–12.06); no other additive interactions were observed.

DISCUSSION

We found that prematurity, in a dose-dependent manner, and congenital malformations were associated with hospitalisations for sepsis and bacterial meningitis in early childhood. Additionally, children born large or small for gestational age were more likely to be hospitalised for sepsis and children exposed to maternal smoking during pregnancy were more likely to be hospitalised for

For peer review only - http://bmjopen.bmj.com/site/about/guidelines.xhtml

bacterial meningitis. Finally, the incidence of sepsis and bacterial meningitis decreased by the end of the study period.

Incidence rates

The incidence rates of sepsis and bacterial meningitis hospitalisations in our study were similar to those reported in previous studies.[1 3 26] During the study period, the incidence rate of pneumococcal meningitis hospitalisations decreased substantially. This was expected, pneumococcal conjugate vaccines were introduced into the Swedish childhood immunisation program between 2007 and 2009.[27] We have previously reported a decreasing incidence of pneumococcal meningitis after 2009 in Västerbotten county (Sweden).[26] No recent national study has examined the incidence of paediatric meningitis in Sweden.

Perinatal factors

In our study, children born prematurely, small for gestational age, with congenital malformations or with male sex were more likely to be hospitalised for sepsis and/or bacterial meningitis. These children are known to be more susceptible to infections in childhood. In a recent Australian cohort study, low gestational age was associated with an increased risk of infection-related deaths and hospital admissions throughout childhood e.g. gestational age <28 weeks was associated with 2.6 times higher risk of invasive bacterial infections.[12] A large Danish cohort study reported an association between low birth weight and infectious disease-related hospitalisations in the first 15 years of life, e.g. children with a birth weight <1000g were 3.3 times more likely to be hospitalised for sepsis.[13] Another Danish study found that congenital malformations and chromosomal abnormalities were associated with respiratory syncytial virus infections in the first 2 years of life.[28] Finally, previous studies have shown that male sex is associated with a large number of childhood infections, including sepsis and meningitis.[3 29 30] Differences in sex hormones has been suggested as one possible explanation, testosterone has an overall suppressive effect on the immune system whereas estrogen

For peer review only

promotes Th1 cellular immune responses and humoral immunity.[29] Overall, these findings are consistent with our results.

In this study, children born large for gestational age were more likely to be hospitalised for sepsis and bacterial meningitis. One explanation could be a higher incidence of maternal diabetes in children born large for gestational age. Both maternal pre-existing diabetes and gestational diabetes are associated with an increased risk of childhood infections.[31] Similar to our results, a national cohort study reported an increased risk for sepsis in children with a birth weight >4500g; however, in contrast to our results, the risk of bacterial meningitis was not increased in children born with high birth weight.[13] Therefore, our findings must be interpreted with caution.

We found an association between maternal smoking during pregnancy and bacterial meningitis. This was anticipated from previous research i.e. children exposed to maternal smoking seems to be at risk of meningococcal infections. In a large cohort study from the UK, children exposed to maternal smoking during pregnancy were more likely to be hospitalised for meningococcal meningitis; no association was found for haemophilus meningitis.[32] Moreover, a US study reported that maternal smoking during pregnancy was associated with 2.9 times higher risk of invasive meningococcal disease in children three years and younger.[30] Maternal smoking during pregnancy is a proxy for maternal smoking after birth.[32] Cigarette smoke may increase the risk of bacterial meningitis by decreasing bronchial ciliary activity, decreasing neutrophil function and by predisposing children to viral respiratory infection.[30]

Numerous studies have shown that social deprivation is associated with an increased risk of disease including paediatric infections In a Danish cohort study, low maternal education level was associated with 1.3 times higher risk of hospitalisation for infectious diseases in the first 5 years of life.[33] A national cohort study from New Zeeland reported a higher incidence of infection-related hospitalisations in children belonging to economically deprived minorities i.e. the Māori and Pacific peoples.[34] Additionally, a US study reported that low maternal education was associated with a two

For peer review only - http://bmjopen.bmj.com/site/about/guidelines.xhtml

times higher risk of invasive meningococcal disease in young children.[30] In our study, low maternal education was associated with an increased risk of hospitalisations for sepsis and bacterial meningitis in the crude analyses; however, these associations decreased and were non-significant in the adjusted analyses. This indicates that the effect of low maternal education was, at least partly, mediated by pregnancy and birth characteristics.

Clinical implications

Pregnancy and birth characteristics are generally considered in clinical guidelines for neonatal infections.[9-11] In contrast, perinatal risk factors are not regularly considered in clinical guidelines for severe infections after the neonatal period, including Swedish and UK guidelines.[7 8 11 14] However, the Swedish guideline recommends that healthcare professionals consider chronic conditions including congenital malformations.[14] We found that prematurity, small or large for gestational age, congenital malformations and maternal smoking during pregnancy were associated with severe infections in young children. Our findings are supported by previous studies.[12 13 28 30 32] Therefore, we suggest that clinical guidelines for risk assessment of infections after the neonatal period consider perinatal factors as well.

Strengths and limitations

Strengths of this study include the use of high-quality national registers with information on a wide range of perinatal factors. This allowed us to systematically examine associations between perinatal factors and hospitalisations for severe paediatric infections. However, our study has several limitations. First, infections were identified using de-identified hospital discharge data. Consequently, cases could not be confirmed by laboratory findings or by other information obtained from medical records. Secondly, we were unable to examine categories of congenital malformations. This is problematic since susceptibility to infections varies between different congenital conditions.[28] Moreover, the observed interaction between prematurity and congenital malformations on the risk of sepsis may be due to more severe malformations in prematurely born children. Third, all children

For peer review only

in Sweden have access to free healthcare and working parents are entitled to paid childcare leave.[35] Consequently, associations between sociodemographic characteristics and severe infections may not be generalised to other settings with less extensive welfare systems. Fourth, our study lacks information on postnatal factors associated with infectious diseases e.g. acquired chronic diseases, vaccination status and breastfeeding. Finally, data was missing for a large proportion (17%) of children. However, results from multiple imputation models were overall consistent with results from the complete case models, indicating no major selection bias.

CONCLUSIONS

In our study, several perinatal factors i.e. prematurity, small for gestational age, large for gestation age, congenital malformations and maternal smoking were associated with an increased risk of sepsis and/or bacterial meningitis in children after the immediate neonatal period up to two years of age. Therefore, we suggest that clinical guidelines for risk assessment of infections in young children consider perinatal factors.

For peer review only

Contributors: SV conceptualised and designed the study together with SAS, carried out analyses and drafted the initial manuscript. UJK contributed to the study design, reviewed and revised the manuscript. TL contributed to the study design, reviewed and revised the manuscript. SAS conceptualised and designed the study together with SV, coordinated and supervised the database, reviewed and revised the manuscript.

Funding: This study was supported by the Unit of Research, Development, and Education, Östersund Hospital, Östersund, Sweden JLL-930202 (to SV); ALF Umeå University, Umeå, Sweden RV-933162 (to SAS).

Competing interests: We have no conflict of interest to declare.

Patient consent: Patient informed consent was not needed because the data were collected retrospectively and anonymised prior to analysis.

Ethics approval: The study was approved by the Regional Ethics Board in Umeå, reference number 2012-265-31M and 2017-399-32M.

Acknowledgement: The authors would like to thank Per E. Gustafsson, PhD at the Department of Epidemiology and Global Health, Umeå University, for reviewing and revising the manuscript and Johan Boström, MD at Department of Clinical Sciences, Pediatrics, Umeå University for help in creating the dataset. Håkan Sjöberg, MEc at Statistics Sweden, for help in compiling the dataset.

Data sharing statement: We used deidentified register data obtained from third parties. It includes sensitive information and some access restrictions may apply. Interested researchers need to obtain data directly from the National Board of Health and Welfare in Sweden (socialstyrelsen@socialstyrelsen.se) and Statistics Sweden (scb@scb.se).

Children included in the study were identified in the Medical Birth Register, data on hospitalisations were obtained from the Swedish National Patient Register and data on deaths were obtained from the Cause of Death Register. All of these registers are maintained by the National Board of Health

For peer review only

and Welfare in Sweden. Data on maternal education were obtained from the Longitudinal Integration Database for Health Insurance and Labour market Studies and data on migration were obtained from The Total Population Register, both registers are maintained by Statistics Sweden.

For peer review only

REFERENCES

1 Fleischmann-Struzek C, Goldfarb DM, Schlattmann P, Schlapbach LJ, Reinhart K, Kissoon N. The global burden of paediatric and neonatal sepsis: a systematic review. *Lancet Respir Med* 2018;6:223-30.
2 Collaborators GBDM. Global, regional, and national burden of meningitis, 1990-2016: a systematic analysis for the Global Burden of Disease Study 2016. *Lancet Neurol* 2018;17:1061-82.
3 Agyeman PKA, Schlapbach LJ, Giannoni E*, et al.* Epidemiology of blood culture-proven bacterial sepsis in children in Switzerland: a population-based cohort study. *Lancet Child Adolesc Health* 2017;1:124-33.
4 Schlapbach LJ, Straney L, Alexander J*, et al.* Mortality related to invasive infections, sepsis, and septic shock in critically ill children in Australia and New Zealand, 2002-13: a multicentre retrospective cohort study. *Lancet Infect Dis* 2015;15:46-54.
5 Ladhani SN, Collins S, Djennad A*, et al.* Rapid increase in non-vaccine serotypes causing invasive pneumococcal disease in England and Wales, 2000-17: a prospective national observational cohort study. *Lancet Infect Dis* 2018;18:441-51.
6 Weiss SL, Peters MJ, Alhazzani W*, et al.* Surviving sepsis campaign international guidelines for the management of septic shock and sepsis-associated organ dysfunction in children. *Intensive care medicine* 2020;46:10-67.
7 van de Beek D, Cabellos C, Dzupova O*, et al.* ESCMID guideline: diagnosis and treatment of acute bacterial meningitis. *Clinical microbiology and infection : the official publication of the European Society of Clinical Microbiology and Infectious Diseases* 2016;22 Suppl 3:S37-62.
8 National Institute for Health and Care Excellence. Fever in under 5s: assessment and initial management. 2019.
9 Puopolo KM, Benitz WE, Zaoutis TE, Committee On F, Newborn, Committee On Infectious D. Management of Neonates Born at >/=35 0/7 Weeks' Gestation With Suspected or Proven Early-Onset Bacterial Sepsis. *Pediatrics* 2018;142.
10 National Institute for Health and Care Excellence. Neonatal infection (early onset): antibiotics for prevention and treatment. 2012.
11 National Institute for Health and Care Excellence. Sepsis: recognition, diagnosis and early management. 2016.
12 Miller JE, Hammond GC, Strunk T*, et al.* Association of gestational age and growth measures at birth with infection-related admissions to hospital throughout childhood: a population-based, data-linkage study from Western Australia. *Lancet Infect Dis* 2016;16:952-61.
13 Hviid A, Melbye M. The impact of birth weight on infectious disease hospitalization in childhood. *American journal of epidemiology* 2007;165:756-61.
14 The Public Health Agency of Sweden. Tecken på allvarlig infektion hos barn. 2014.
15 The National Board of Health and Welfare in Sweden. The Swedish Medical Birth Register - A summary of content and quality. 2003.
16 Ludvigsson JF, Andersson E, Ekbom A*, et al.* External review and validation of the Swedish national inpatient register. *BMC public health* 2011;11:450.
17 Ludvigsson JF, Svedberg P, Olen O, Bruze G, Neovius M. The longitudinal integrated database for health insurance and labour market studies (LISA) and its use in medical research. *European journal of epidemiology* 2019;34:423-37.
18 Brooke HL, Talback M, Hornblad J*, et al.* The Swedish cause of death register. *European journal of epidemiology* 2017;32:765-73.
19 Ludvigsson JF, Lu D, Hammarstrom L, Cnattingius S, Fang F. Small for gestational age and risk of childhood mortality: A Swedish population study. *PLoS medicine* 2018;15:e1002717.
20 The National Board of Health and Welfare in Sweden. Registration of Congenital Malformations in the Swedish Health Registers. 2004.

For peer review only

21 Mattsson K, Kallen K, Rignell-Hydbom A*, et al.* Cotinine Validation of Self-Reported Smoking During Pregnancy in the Swedish Medical Birth Register. *Nicotine & tobacco research : official journal of the Society for Research on Nicotine and Tobacco* 2016;18:79-83.
22 Hanley JA, Negassa A, Edwardes MD, Forrester JE. Statistical analysis of correlated data using generalized estimating equations: an orientation. *American journal of epidemiology* 2003;157:364-75.
23 Hosmer DW, Lemeshow S. Confidence interval estimation of interaction. *Epidemiology* 1992;3:452-6.
24 VanderWeele TJ, Knol MJ. A Tutorial on Interaction. *Epidemiologic Methods* 2014;3:33-72.
25 Sterne JA, White IR, Carlin JB*, et al.* Multiple imputation for missing data in epidemiological and clinical research: potential and pitfalls. *Bmj* 2009;338:b2393.
26 Johansson Kostenniemi U, Norman D, Sellin M, Silfverdal SA. Sustained reductions of invasive infectious disease following general infant Haemophilus influenzae type b and pneumococcal vaccination in a Swedish Arctic region. *Acta paediatrica* 2019;108:1871-8.
27 Berglund A, Ekelund M, Fletcher MA, Nyman L. All-cause pneumonia hospitalizations in children <2 years old in sweden, 1998 to 2012: impact of pneumococcal conjugate vaccine introduction. *PloS one* 2014;9:e112211.
28 Kristensen K, Hjuler T, Ravn H, Simoes EA, Stensballe LG. Chronic diseases, chromosomal abnormalities, and congenital malformations as risk factors for respiratory syncytial virus hospitalization: a population-based cohort study. *Clinical infectious diseases : an official publication of the Infectious Diseases Society of America* 2012;54:810-7.
29 Muenchhoff M, Goulder PJ. Sex differences in pediatric infectious diseases. *The Journal of infectious diseases* 2014;209 Suppl 3:S120-6.
30 Yusuf HR, Rochat RW, Baughman WS*, et al.* Maternal cigarette smoking and invasive meningococcal disease: a cohort study among young children in metropolitan Atlanta, 1989-1996. *American journal of public health* 1999;89:712-7.
31 Aberg A, Westbom L. Association between maternal pre-existing or gestational diabetes and health problems in children. *Acta paediatrica* 2001;90:746-50.
32 Goldacre MJ, Wotton CJ, Maisonneuve JJ. Maternal and perinatal factors associated with subsequent meningococcal, Haemophilus or enteroviral meningitis in children: database study. *Epidemiology and infection* 2014;142:371-8.
33 Biering-Sorensen S, Sondergaard G, Vitting Andersen K, Andersen AM, Mortensen LH. Time trends in socio-economic factors and risk of hospitalisation with infectious diseases in pre-school children 1985-2004: a Danish register-based study. *Paediatric and perinatal epidemiology* 2012;26:226-35.
34 Baker MG, Barnard LT, Kvalsvig A*, et al.* Increasing incidence of serious infectious diseases and inequalities in New Zealand: a national epidemiological study. *Lancet* 2012;379:1112-9.
35 Wettergren B, Blennow M, Hjern A, Soder O, Ludvigsson JF. Child Health Systems in Sweden. *The Journal of pediatrics* 2016;177S:S187-S202.

For peer review only

Table 1: Birth, pregnancy and sociodemographic characteristics of the study population

	Included			Excluded*
	Sepsis	Bacterial meningitis	All included	
	n = 1011	n = 382	n = 1 406 547	n = 280 202
Sex:				
Male	55.8 (564)	57.9 (221)	51.4 (722 988)	51.4 (144 079)
Female	44.2 (447)	42.4 (162)	48.6 (683 559)	48.6 (136 117)
Gestation age†:				
Extremely preterm	2.5 (25)	1.0 (4)	0.1 (2012)	0.7 (1927)
Very preterm	2.4 (24)	2.1 (8)	0.4 (5457)	1.6 (4418)
Moderate preterm	8.8 (89)	5.8 (22)	4.0 (56 291)	10.3 (28 979)
Term	80.7 (816)	84.8 (324)	88.3 (1 242 234)	81.4 (227 950)
Post-term	5.6 (57)	6.5 (25)	7.1 (100 553)	5.7 (15 926)
SGA:				
Yes	7.8 (79)	3.7 (14)	2.1 (30 099)	2.1 (5972)
No	92.2 (932)	96.6 (369)	97.9 (1 376 448)	78.7 (220 473)
LGA:				
Yes	5.1 (52)	5.0 (19)	3.7 (51 816)	3.0 (8334)
No	94.9 (959)	95.3 (364)	96.3 (1 354 731)	77.8 (218 111)
Congenital malformation‡:				
Yes	14.1 (143)	6.0 (23)	3.4 (48 209)	3.7 (10 401)
No	85.9 (868)	94.2 (360)	96.6 (1 358 338)	96.3 (269 801)
Maternal age:				
Mean (SD)	30.0 (5.1)	29.7 (5.5)	30.1 (5.1)	30.0 (5.5)
Maternal smoking:				
No smoking	90.6 (916)	86.9 (332)	91.5 (1 286 708)	61.3 (171 790)
Smoking	9.4 (95)	13.4 (51)	8.5 (119 839)	6.9 (19 472)
Pregnancy BMI§:				
Underweight	2.1 (21)	2.4(9)	2.4 (33 836)	1.0 (2895)
Normal	59.8 (605)	60.7 (232)	61.6 (865 874)	20.3 (56 838)
Overweight	25.7 (260)	25.1 (96)	24.8 (348 695)	9.1 (25 600)
Obese	12.4 (125)	12.0 (46)	11.2 (158 142)	4.3 (12 030)
Parity:				
Mean (SD)	1.9 (1.0)	1.8 (1.1)	1.8 (1.0)	2.0 (1.2)
Maternal education in years:				
≤9	14.8 (150)	15.4 (59)	11.6 (162 814)	10.6 (29 682)
10–12	42.0 (425)	42.4 (162)	43.6 (613 370)	36.6 (102 593)
13–14	12.7 (127)	16.0 (61)	13.7 (192 659)	12.6 (35 362)
≥15	30.6 (309)	26.4 (101)	31.1 (437 704)	25.7 (71 907)
Maternal country of birth:				
Sweden	77.3 (782)	80.6 (308)	81.2 (1 142 682)	70.9 (198 735)
Other Nordic	1.8 (18)	1.8 (7)	1.7 (23 403)	2.6 (7173)
Other Europe and North America	8.8 (89)	6.8 (26)	6.2 (87 864)	9.0 (25 278)
Asia	8.6 (87)	6.3 (24)	7.4 (103 858)	10.8 (30 392)

For peer review only

Africa	2.4 (24)	3.4 (13)	2.4 (33 230)	5.2 (14 644)
Other	1.1 (11)	1.3 (5)	1.1 (15 510)	1.3 (3688)
Year of birth:				
1997–2002	27.1 (274)	33.0 (126)	29.5 (41 4783)	40.3 (112 908)
2003–2008	40.6 (410)	46.1 (176)	35.8 (503 841)	37.1 (103 988)
2009–2013	32.3 (327)	21.2 (81)	34.7 (487 923)	22.6 (63 306)

Values are percentages (numbers) unless stated otherwise.
*Children with missing data were excluded (17% of the original cohort). Data on variables were available in 35%–100% of the excluded children.
†Gestational age categorised as extremely premature (22–27 weeks), very premature (28–31 weeks), moderate premature (32–36 weeks), term (37–41 weeks) and post-term (≥42 weeks).
‡ICD-10 codes: Q00–Q99.
§BMI categorised as underweight (BMI<18.5 kg/m^2). normal (BMI 18.5–24.9 kg/m^2). overweight (BMI 25.0–29.9 kg/ m^2) and obese (BMI≥30.0 kg/m^2).
SGA, small for gestational age. LGA, large for gestational age. BMI, body mass index. SD, standard deviation.
ICD-10, International Classification of Diseases, 10th revision.

For peer review only

Table 2: Associations between perinatal factors and hospitalisations for sepsis and bacterial meningitis in early childhood

	Sepsis		Bacterial meningitis	
	Crude IRR (95% CI)	Adjusted IRR (95% CI)	Crude IRR (95% CI)	Adjusted IRR (95% CI)
Sex:				
Male	1.12 (0.97–1.30)	1.09 (0.94–1.26)	1.35 (1.09–1.66)	1.33 (1.08–1.64)
Female	1 ref	1 ref	1 ref	1 ref
Gestational age*:				
Extremely preterm	15.73 (10.49–23.59)	10.37 (6.78–15.86)	7.50 (2.80–20.10)	6.22 (2.28–16.94)
Very preterm	6.44 (4.07–10.18)	4.06 (2.49–6.63)	5.99 (2.89–12.45)	4.95 (2.39–10.24)
Moderate preterm	2.17 (1.71–2.76)	1.85 (1.45–2.37)	1.41 (0.92–2.18)	1.31 (0.84–2.03)
Term	1 ref	1 ref	1 ref	1 ref
Post-term	0.80 (0.59–1.07)	0.80 (0.59–1.08)	0.93 (0.62–1.41)	0.90 (0.59–1.36)
SGA:				
Yes	3.51 (2.72–4.53)	2.44 (1.83–3.25)	1.89 (1.08–3.30)	1.36 (0.78–2.40)
No	1 ref	1 ref	1 ref	1 ref
LGA:				
Yes	1.69 (1.18–2.42)	1.71 (1.19–2.44)	1.43 (0.89–2.31)	1.51 (0.93–2.45)
No	1 ref	1 ref	1 ref	1 ref
Congenital malformation†:				
Yes	4.41 (3.61–5.40)	3.89 (3.17–4.77)	1.86 (1.21–2.88)	1.69 (1.09–2.62)
No	1 ref	1 ref	1 ref	1 ref
Maternal age:				
Per year of age	0.99 (0.98–1.01)	0.99 (0.97–1.00)	0.99 (0.96–1.01)	0.99 (0.97–1.02)
Maternal smoking:				
Yes	1.08 (0.85–1.38)	0.96 (0.75–1.25)	1.65 (1.22–2.24)	1.46 (1.05–2.04)
No	1 ref	1 ref	1 ref	1 ref
Pregnancy BMI‡:				
Underweight	0.84 (0.52–1.37)	0.77 (0.48–1.26)	0.95 (0.49–1.85)	0.88 (0.45–1.71)
Normal	1 ref	1 ref	1 ref	1 ref

For peer review only

Overweight	1.13 (0.95–1.33)	1.10 (0.92–1.30)	1.08 (0.84–1.39)	1.06 (0.82–1.36)
Obese	1.21 (0.96–1.52)	1.11 (0.88–1.39)	1.06 (0.77–1.46)	0.99 (0.72–1.37)
Parity:				
Per child	1.05 (0.98–1.13)	1.08 (1.00–1.16)	0.98 (0.87–1.10)	0.96 (0.85–1.08)
Maternal education in years:				
≤9	1.27 (1.01–1.59)	1.07 (0.83–1.39)	1.70 (1.23–2.36)	1.39 (0.95–2.02)
10–12	0.95 (0.80–1.13)	0.89 (0.74–1.07)	1.23 (0.96–1.59)	1.09 (0.83–1.43)
13–14	0.93 (0.73–1.18)	0.90 (0.71–1.15)	1.46 (1.06–2.02)	1.37 (0.98–1.90)
≥15	1 ref	1 ref	1 ref	1 ref
Maternal country of birth:				
Sweden	1 ref	1 ref	1 ref	1 ref
Other Nordic	1.03 (0.62–1.72)	1.01 (0.61–1.69)	1.23 (0.56–2.70)	1.18 (0.54–2.58)
Other Europe and North America	1.36 (1.07–1.72)	1.33 (1.05–1.70)	1.09 (0.73–1.64)	1.10 (0.73–1.66)
Asia	1.24 (0.96–1.61)	1.17 (0.90–1.53)	0.92 (0.60–1.42)	0.94 (0.61–1.46)
Africa	1.12 (0.65–1.94)	1.00 (0.57–1.75)	1.50 (0.84–2.65)	1.60 (0.91–2.80)
Other	0.88 (0.47–1.62)	0.85 (0.46–1.57)	1.37 (0.54–3.49)	1.38 (0.54–3.52)
Year of birth:				
1997–2002	1 ref	1 ref	1 ref	1 ref
2003–2008	1.26 (1.05–1.50)	1.23 (1.03–1.48)	1.18 (0.93–1.49)	1.23 (0.96–1.57)
2009–2013	1.00 (0.83–1.20)	0.98 (0.80–1.19)	0.54 (0.41–0.72)	0.57 (0.42–0.77)
Observations (N):	1 406 547	1 406 547	1 406 547	1 406 547
Clusters (Maternal ID):	891 950	891 950	891 950	891 950

Analyses excluded children with missing data (n = 280 202), leaving 1 406 547 children. Adjusted analyses were controlled for sex, gestational age, SGA, LGA, congenital malformation, maternal age, smoking during pregnancy, pregnancy BMI, parity, maternal education level, maternal country of birth and year of birth.

*Gestational age categorised as extremely premature (22–27 weeks), very premature (28–31 weeks), moderate premature (32–36 weeks), term (37–41 weeks) and post-term (≥42 weeks).

†ICD-10 codes: Q00–Q99.

‡BMI categorised as underweight (BMI<18.5 kg/m^2), normal (BMI 18.5–24.9 kg/m^2), overweight (BMI 25.0–29.9 kg/m^2) and obese (BMI≥30.0 kg/m^2).

IRR, incidence rate ratio. CI, confidence interval. SGA, small for gestational age. LGA, large for gestational age. BMI, body mass index. ICD-10, International Classification of Diseases, 10th revision.

Table 3: Interactions between sex, prematurity and perinatal factors

	Sepsis	Bacterial meningitis
RERI with sex*:	RERI (95% CI)	RERI (95% CI)
Prematurity*sex	-0.78 (-1.78–0.22)	1.14 (-0.24–2.53)
SGA*sex	-0.45 (-1.87–0.95)	1.83 (-0.08–3.73)
LGA*sex	0.04 (-1.20–1.27)	1.11 (-0.49–2.71)
Congenital malformations*sex	-0.46 (-2.06–1.13)	-0.96 (-2.80–0.88)
Maternal smoking*sex	-0.56 (-1.37–0.25)	0.07 (-0.61–0.75)
RERI with prematurity†:		
SGA*prematurity	1.00 (-1.36–3.36)	0.36 (-2.73–3.45)
LGA*prematurity	-0.97 (-3.01–1.08)	1.58 (-2.76–5.91)
Congenital malformations*prematurity	7.75 (3.45–12.06)	-0.33 (-3.19–2.53)
Maternal smoking*prematurity	-0.51 (-2.12–1.11)	-0.09 (-2.33–2.16)

Analyses excluded children with missing data (n = 280 202), leaving 1 406 547 children. Analyses were controlled for sex, gestational age, SGA, LGA, congenital malformation, maternal age, smoking during pregnancy, pregnancy BMI, parity, maternal education level, maternal country of birth and year of birth. Gestational age was included as a binary variable (0 = gestational age ≥37 weeks, 1 = gestational age <37 weeks); other variables were included as previously described.

*Female is the reference category.

†Gestational age ≥37 weeks is the reference category.

RERI, relative excess risk due to interaction. CI, confidence interval. SGA, small for gestational age. LGA, large for gestational age.

For peer review only

Figure 1. Flowchart of the study population. Complete case analyses included all children in the study population. Multiple imputation analyses included all children in the target population. *Children with missing data on any variable (sex, gestational age, small for gestational age, large for gestational age, congenital malformation, maternal age, smoking during pregnancy, pregnancy body mass index, parity, maternal education level, maternal country of birth and year of birth) were excluded.

Figure 2. Incidence of sepsis (brown circles) and bacterial meningitis (orange squares) in early childhood, by age in months. Incidence rates calculated as the number of hospital admissions per 100 000 person-years at risk. Vertical lines represent 95% confidence intervals. Analyses included 1 406 547 children.

Figure 3. Incidence of sepsis (brown circles) and bacterial meningitis (orange squares) in early childhood, by year of birth. Incidence rates calculated as the number of hospital admissions per 100 000 person-years at risk. Vertical lines represent 95% confidence intervals. Analyses included 1 406 547 children.

For peer review only

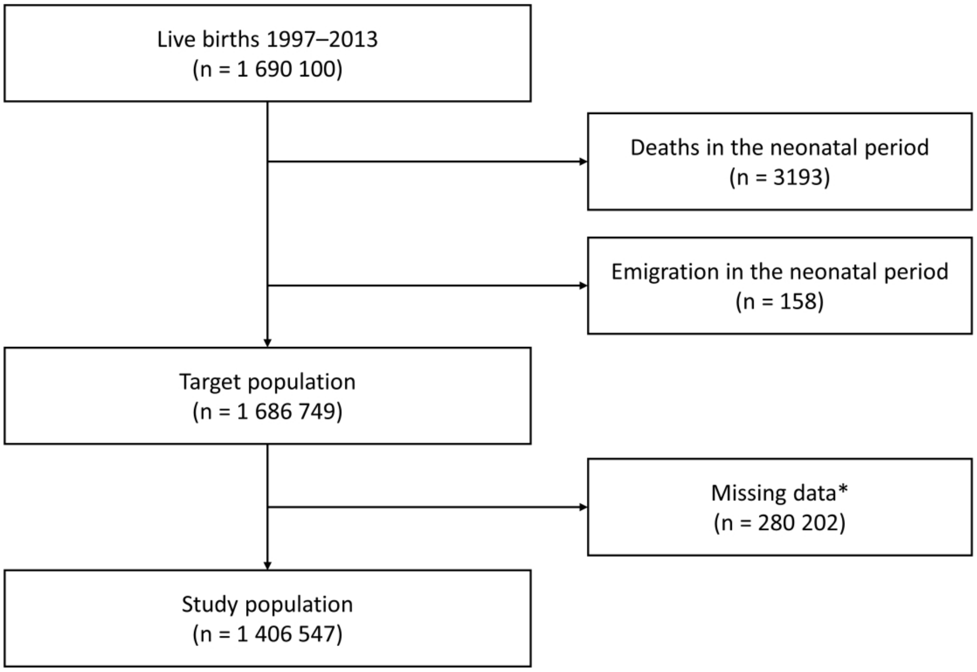

Figure 1. Flowchart of the study population. Complete case analyses included all children in the study population. Multiple imputation analyses included all children in the target population. *Children with missing data on any variable (sex, gestational age, small for gestational age, large for gestational age, congenital malformation, maternal age, smoking during pregnancy, pregnancy body mass index, parity, maternal education level, maternal country of birth and year of birth) were excluded.

2021x1417mm (38 x 38 DPI)

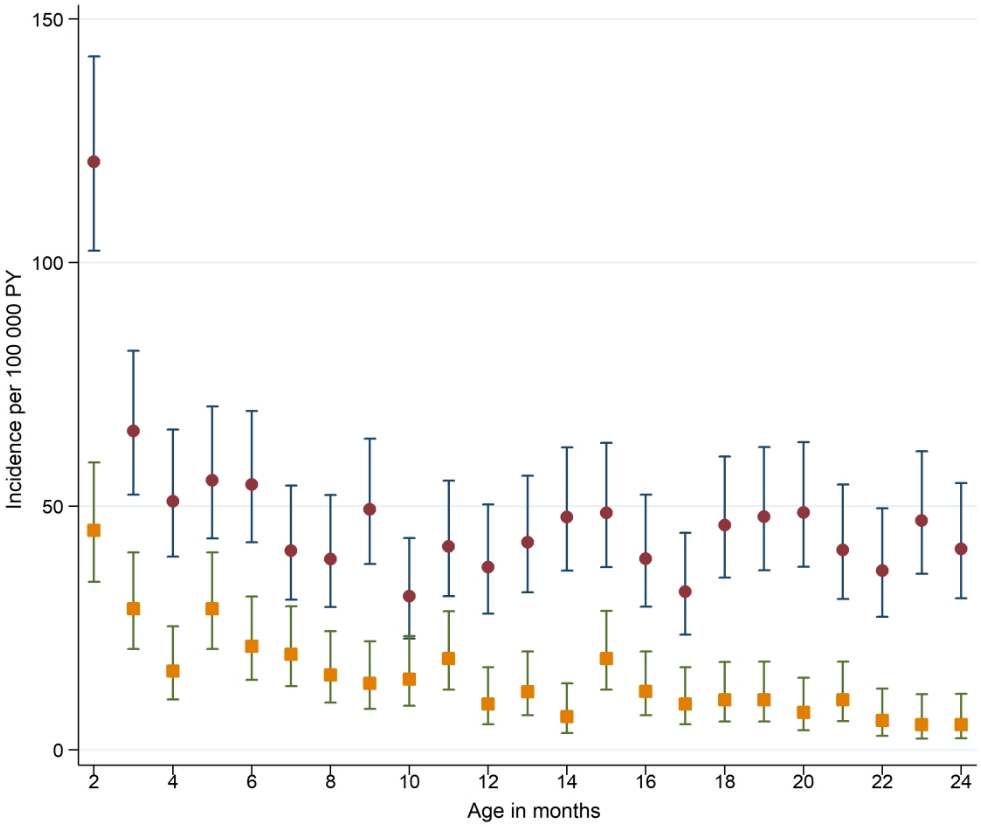

Figure 2. Incidence of sepsis (brown circles) and bacterial meningitis (orange squares) in early childhood, by age in months. Incidence rates calculated as the number of hospital admissions per 100 000 person-years at risk. Vertical lines represent 95% confidence intervals. Analyses included 1 406 547 children.

1909x1593mm (38 x 38 DPI)

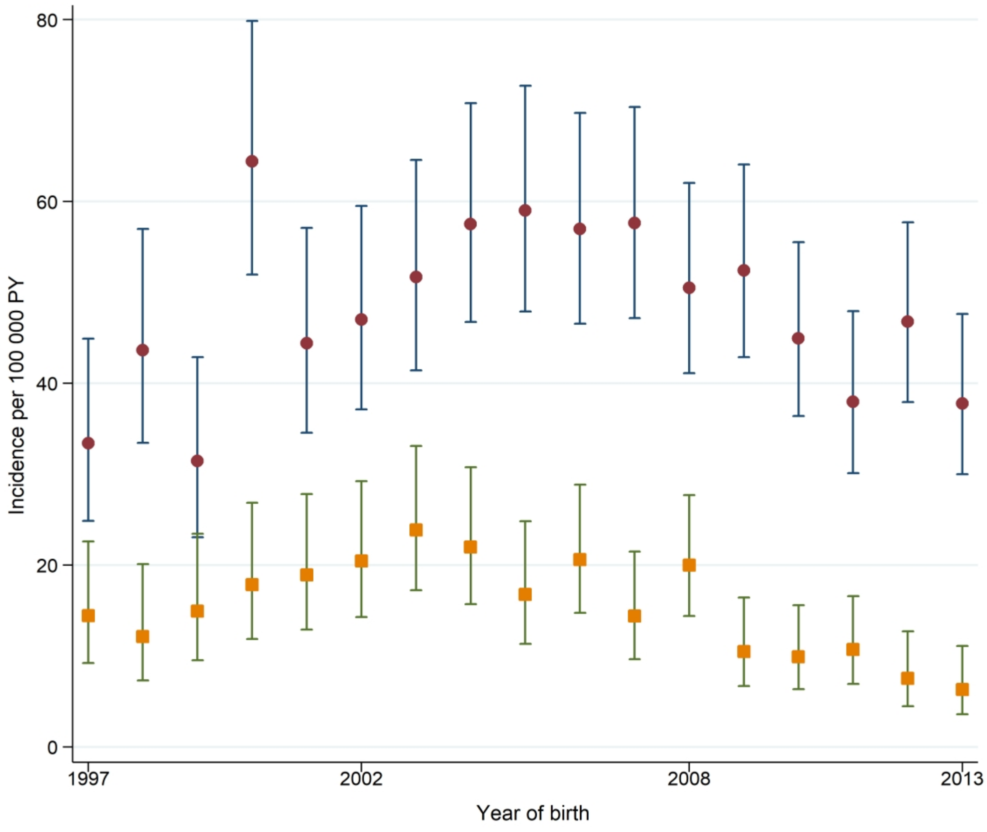

Figure 3. Incidence of sepsis (brown circles) and bacterial meningitis (orange squares) in early childhood, by year of birth. Incidence rates calculated as the number of hospital admissions per 100 000 person-years at risk. Vertical lines represent 95% confidence intervals. Analyses included 1 406 547 children.

1919x1620mm (38 x 38 DPI)

Appendix A

Table A: Incidence overall and for categories of bacterial meningitis in early childhood (28 days until 2 years of age), by year of birth

	1997–2002<br>Incidence (95% CI)	2003–2008<br>Incidence (95% CI)	2009–2013<br>Incidence (95% CI)
All	16.6 (14.0–19.7)	19.6 (17.0–22.6)	9.0 (7.2–11.1)
Meningococcal meningitis*	2.5 (1.6–3.9)	1.6 (0.9–2.6)	1.2 (0.7–2.1)
Haemophilus meningitis†	1.0 (0.5–2.0)	1.0 (0.6–1.9)	0.7 (0.4–1.6)
Pneumococcal meningitis‡	8.3 (6.5–10.6)	10.0 (8.2–12.2)	2.2 (1.5–3.4)
Streptococcus meningitis§	1.5 (0.9–2.7)	1.7 (1.0–2.7)	1.5 (0.9–2.5)
Other meningitis¶	3.3 (2.2–4.8)	5.3 (4.0–6.9)	3.3 (2.3–4.7)

Incidence rates estimated as the number of hospital admissions per 100 000 person-years at risk.
Analyses included 1 406 547 children.
*ICD-10 code A39.0
†ICD-10 code G00.0
‡ICD-10 code G00.1
§ICD-10 code G00.2
¶ICD-10 codes G00.3-G00.8 and A32.1
CI, confidence intervals. ICD-10, International Classification of Diseases, 10th revision.

For peer review only

Appendix B

Table A: Crude and adjusted analyses of the association between perinatal factors and hospitalisations for sepsis and bacterial meningitis in early childhood (28 days until 2 years of age), estimated using multiple imputation

	Sepsis		Bacterial meningitis	
	Crude IRR (95% CI)	Adjusted IRR (95% CI)	Crude IRR (95% CI)	Adjusted IRR (95% CI)
Sex:				
Male	1.07 (0.94–1.22)	1.04 (0.91–1.18)	1.38 (1.14–1.66)	1.36 (1.13–1.64)
Female	1 ref	1 ref	1 ref	1 ref
Gestational age*:				
Extremely preterm	14.83 (10.84–20.30)	9.91 (7.09–13.84)	13.19 (7.23–24.07)	10.85 (5.77–20.41)
Very preterm	5.90 (4.24–8.22)	3.93 (2.74–5.63)	5.06 (2.87–8.94)	4.03 (2.27–7.16)
Moderate preterm	2.50 (2.01–3.10)	2.16 (1.73–2.69)	2.00 (1.45–2.76)	1.84 (1.33–2.55)
Term	1 ref	1 ref	1 ref	1 ref
Post-term	0.80 (0.61–1.04)	0.80 (0.61–1.05)	0.92 (0.62–1.34)	0.88 (0.60–1.30)
SGA:				
Yes	3.47 (2.75–4.37)	2.18 (1.69–2.83)	2.53 (1.61–3.97)	1.63 (1.02–2.62)
No	1 ref	1 ref	1 ref	1 ref
LGA:				
Yes	1.61 (1.17–2.20)	1.61 (1.17–2.21)	1.29 (0.83–2.01)	1.36 (0.87–2.13)
No	1 ref	1 ref	1 ref	1 ref
Congenital malformation†:				
Yes	4.68 (3.95–5.55)	3.96 (3.32–4.71)	2.01 (1.40–2.89)	1.70 (1.18–2.45)
No	1 ref	1 ref	1 ref	1 ref
Maternal age:				
Per year of age	0.99 (0.98–1.00)	0.98 (0.97–1.00)	0.98 (0.97–1.00)	0.99 (0.97–1.01)
Maternal smoking:				
Yes	1.16 (0.94–1.45)	1.00 (0.79–1.26)	1.68 (1.27–2.23)	1.47 (1.08–2.01)
No	1 ref	1 ref	1 ref	1 ref

For peer review only

Pregnancy BMI‡:				
Underweight	0.81 (0.51–1.29)	0.73 (0.46–1.16)	0.90 (0.46–1.74)	0.81 (0.42–1.57)
Normal	1 ref	1 ref	1 ref	1 ref
Overweight	1.12 (0.96–1.32)	1.09 (0.93–1.28)	1.09 (0.85–1.39)	1.05 (0.83–1.35)
Obese	1.24 (1.00–1.53)	1.11 (0.90–1.38)	1.07 (0.79–1.45)	0.98 (0.72–1.34)
Parity:				
Per child	1.06 (1.01–1.12)	1.09 (1.02–1.15)	1.01 (0.92–1.11)	1.00 (0.90–1.10)
Maternal education in years:				
≤9	1.41 (1.15–1.72)	1.15 (0.90–1.46)	1.72 (1.29–2.29)	1.33 (0.95–1.87)
10–12	1.00 (0.85–1.17)	0.91 (0.77–1.09)	1.23 (0.98–1.55)	1.07 (0.83–1.37)
13–14	0.96 (0.77–1.18)	0.93 (0.75–1.16)	1.42 (1.06–1.89)	1.33 (0.99–1.78)
≥15	1 ref	1 ref	1 ref	1 ref
Maternal country of birth:				
Sweden	1 ref	1 ref	1 ref	1 ref
Other Nordic	1.22 (0.79–1.91)	1.20 (0.76–1.87)	1.13 (0.57–2.25)	1.08 (0.54–2.14)
Other Europe and North America	1.43 (1.16–1.76)	1.39 (1.13–1.70)	1.10 (0.78–1.56)	1.08 (0.76–1.53)
Asia	1.20 (0.96–1.49)	1.10 (0.88–1.38)	0.97 (0.68–1.40)	0.97 (0.67–1.39)
Africa	1.20 (0.82–1.75)	1.03 (0.69–1.53)	1.54 (0.98–2.41)	1.57 (1.00–2.46)
Other	0.99 (0.56–1.75)	0.96 (0.54–1.70)	1.93 (0.96–3.87)	1.91 (0.95–3.86)
Year of birth:				
1997–2002	1 ref	1 ref	1 ref	1 ref
2003–2008	1.20 (1.02–1.40)	1.20 (1.02–1.41)	1.23 (1.00–1.52)	1.29 (1.04–1.59)
2009–2013	1.00 (0.85–1.18)	1.00 (0.84–1.19)	0.58 (0.45–0.75)	0.61 (0.47–0.80)
Observations (N):	1 406 547	1 406 547	1 406 547	1 406 547
Clusters (Maternal ID):	891 950	891 950	891 950	891 950

Multiple imputation models included all 1 686 749 children. Adjusted analyses were controlled for sex, gestational age, small for gestational age, large for gestational age, congenital malformation, maternal age, smoking during pregnancy, pregnancy BMI, parity, maternal education level, maternal country of birth and year of birth.

For peer review only

*Gestational age categorised as extremely premature (22–27 weeks), very premature (28–31 weeks), moderate premature (32–36 weeks), term (37–41 weeks) and post-term (≥42 weeks)
†ICD-10 codes: Q00–Q99.
‡BMI categorised as underweight (BMI<18.5 kg/m^2), normal (BMI 18.5–24.9 kg/m^2), overweight (BMI 25.0–29.9 kg/m^2) and obese (BMI≥30.0 kg/m^2).
IRR, incidence rate ratio. CI, confidence intervals. SGA, small for gestational age. LGA, large for gestational age. BMI, body mass index. ICD-10, International Classification of Diseases, 10th revision.

For peer review only

STROBE Statement—Checklist of items that should be included in reports of ***cohort studies***

	Item No	Recommendation	Page No
**Title and abstract**	1	(*a*) Indicate the study's design with a commonly used term in the title or the abstract	1
		(*b*) Provide in the abstract an informative and balanced summary of what was done and what was found	2
**Introduction**			
Background/rationale	2	Explain the scientific background and rationale for the investigation being reported	4
Objectives	3	State specific objectives, including any prespecified hypotheses	4
**Methods**			
Study design	4	Present key elements of study design early in the paper	5
Setting	5	Describe the setting, locations, and relevant dates, including periods of recruitment, exposure, follow-up, and data collection	5
Participants	6	(*a*) Give the eligibility criteria, and the sources and methods of selection of participants. Describe methods of follow-up	5 + fig 1
		(*b*) For matched studies, give matching criteria and number of exposed and unexposed	
Variables	7	Clearly define all outcomes, exposures, predictors, potential confounders, and effect modifiers. Give diagnostic criteria, if applicable	5-6
Data sources/ measurement	8*	For each variable of interest, give sources of data and details of methods of assessment (measurement). Describe comparability of assessment methods if there is more than one group	5-6
Bias	9	Describe any efforts to address potential sources of bias	7
Study size	10	Explain how the study size was arrived at	-
Quantitative variables	11	Explain how quantitative variables were handled in the analyses. If applicable, describe which groupings were chosen and why	5-6
Statistical methods	12	(*a*) Describe all statistical methods, including those used to control for confounding	6-7
		(*b*) Describe any methods used to examine subgroups and interactions	
		(*c*) Explain how missing data were addressed	
		(*d*) If applicable, explain how loss to follow-up was addressed	
		(*e*) Describe any sensitivity analyses	
**Results**			
Participants	13*	(a) Report numbers of individuals at each stage of study—eg numbers potentially eligible, examined for eligibility, confirmed eligible, included in the study, completing follow-up, and analysed	7-8
		(b) Give reasons for non-participation at each stage	
		(c) Consider use of a flow diagram	
Descriptive data	14*	(a) Give characteristics of study participants (eg demographic, clinical, social) and information on exposures and potential confounders	8 + Table 1
		(b) Indicate number of participants with missing data for each variable of interest	Table 1
		(c) Summarise follow-up time (eg, average and total amount)	8
Outcome data	15*	Report numbers of outcome events or summary measures over time	8

For peer review only - http://bmjopen.bmj.com/site/about/guidelines.xhtml

Main results	16	(*a*) Give unadjusted estimates and, if applicable, confounder-adjusted estimates and their precision (eg, 95% confidence interval). Make clear which confounders were adjusted for and why they were included<br>(*b*) Report category boundaries when continuous variables were categorized<br>(*c*) If relevant, consider translating estimates of relative risk into absolute risk for a meaningful time period	8-9<br>Table2
Other analyses	17	Report other analyses done—eg analyses of subgroups and interactions, and sensitivity analyses	Table3 Appendix B
**Discussion**			
Key results	18	Summarise key results with reference to study objectives	9
Limitations	19	Discuss limitations of the study, taking into account sources of potential bias or imprecision. Discuss both direction and magnitude of any potential bias	11-12
Interpretation	20	Give a cautious overall interpretation of results considering objectives, limitations, multiplicity of analyses, results from similar studies, and other relevant evidence	9-11
Generalisability	21	Discuss the generalisability (external validity) of the study results	12
**Other information**			
Funding	22	Give the source of funding and the role of the funders for the present study and, if applicable, for the original study on which the present article is based	13

*Give information separately for exposed and unexposed groups.

Note: An Explanation and Elaboration article discusses each checklist item and gives methodological background and published examples of transparent reporting. The STROBE checklist is best used in conjunction with this article (freely available on the Web sites of PLoS Medicine at http://www.plosmedicine.org/, Annals of Internal Medicine at http://www.annals.org/, and Epidemiology at http://www.epidem.com/). Information on the STROBE Initiative is available at http://www.strobe-statement.org.

For peer review only
